# When a Low-Temperature Solid Oxide Solution Is the Best Solution: Inserting Guest Ions into the Ceria Host Lattice to Improve Silver Catalyst Activity for Soot Combustion

**DOI:** 10.1021/acsami.5c21439

**Published:** 2026-03-06

**Authors:** Ewa M. Iwanek nee Wilczkowska

**Affiliations:** Faculty of Chemistry, 49566 Warsaw University of Technology, Noakowskiego 3, 00-664 Warsaw, Poland

**Keywords:** heterogeneous catalyst, ceria-based support, soot combustion, silver catalyst, ToF-SIMS

## Abstract

This study focuses on the application of low-temperature ceria-based solid solutions as silver supports for the catalytic soot combustion. The silver catalysts supported on the solid solutions exhibited activity substantially higher than that on undoped ceria. Although ceria has often been investigated as a catalyst or catalytic support, the present study shows that the proper choice of dopant can further increase the activity of the ceria supports themselves as well as the resulting silver catalysts. This investigation can be a breakthrough in the study of the impact of the selection of the support dopant for a beneficial overall effect of the use of low-temperature solid solutions as supports. All of the synthesized supports contain only one phase, i.e., the fluorite-type structure, typical for undoped ceria with the same lattice parameter and the same dopant concentration. The influence of support composition on the crystallite size (X-ray diffraction, XRD), shape of pores (N_2_ physisorption), acid/base properties (Py-FTIR, Hammett indicator tests), and concentration of oxygen vacancies (Raman) was investigated. The distribution of 5 atom % of Sr^2+^, Mg^2+^, Al^3+^, or Zr^4+^ in the ceria lattice as well as the distribution of silver on these supports was probed with energy-dispersive X-ray (EDX) and ToF SIMS. Elemental maps show an even distribution of the dopants within the grains of the supports. The silver was mainly present as a metallic silver layer on the supports, though the systems exhibited differences in the concentrations of oxidized silver species on their surface and in the reducibility of the supports, as evidenced by H_2_–temperature-programmed desorption studies.

## Graphical abstract

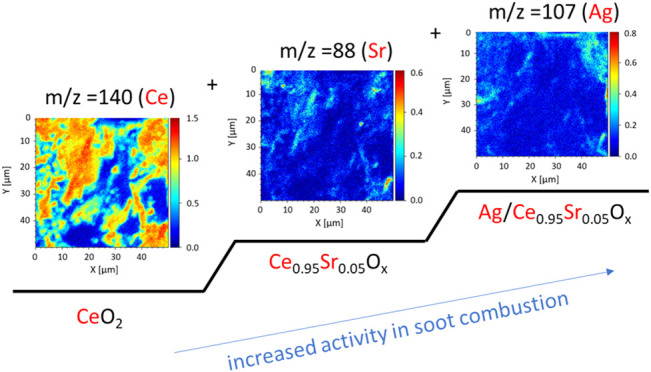

## Introduction

Highly sintered, low surface area ceria-based solid solutions are one of the best studied types of doped oxides due to their application as solid electrolytes in solid oxide fuel cells.^1–3^ Several ions have been used to dope ceria, such as Gd^3+^, Sm^3+^, and Sr^2+^.^4–8^ The ion most frequently introduced into the CeO_2_ cation sublattice for this particular purpose is Gd^3+^.^4,5^ In the case of SOFCs, a similar ionic radius of the dopants (Sm^3+^ and Gd^3+^) and Ce^4+^ is beneficial for its ionic conductivity.^6,7^ However, the ionic size may not be the most important parameter for supports of a silver catalyst in soot oxidation. Doping ceria with strontium ions has also been investigated.^3,8^ In one study, a series of solid oxide solutions with the formula Ce_1–*x*_Sr_*x*_O_2−δ_ were synthesized by calcining powders at a temperature of 1450 °C, and at *x* = 0.1, peaks from a second phase, namely, SrCeO_3_, were observed in the diffraction pattern. A similar finding was reported by Jaiswal et al. in a study in which the following compositions were tested: Ce_1–*x*_Sr_*x*_O_2–*x*_ with 0.05 ≤ *x* ≤ 0.20, which involved calcination at 1350 °C for 4 h. It was noted that the formation of SrCeO_3_ led to a decrease in the ionic conductivity of the solid electrolyte.^6^ The high thermal treatment is most likely the reason for the formation of this compound. The best ionic conductivity was noted for 5 atom % of Sr^2+^ inserted into the ceria lattice. In light of the change in the conductivity of cerium oxide under the influence of doping with other ions, the question arises as to how doping of cerium oxide affects its catalytic activity or, when used as a support, the overall activity of the catalyst. For purposes other than obtaining catalysts, a few studies on the implementation of Sr doping in a variety of solids have been conducted. Their aim was to widen the band gap of solids such as HfO_2_, ZnO, and CeO_2_.^9–11^

The ceria-based electrolytes used in SOFCs are obtained at very high temperatures and are therefore not effective for the role of catalytic supports, in which a high surface area is desirable. This study focuses on low-temperature solid oxide solutions in which foreign ions are introduced into the ceria lattice during the formation of ceria from a coprecipitated precursor rather than by sintering oxides together. By itself, or with a dopant, ceria crystallizes in the fluorite-type structure.^12,13^ The only differences between the obtained structures can be (1) the lattice parameter, (2) concentration of foreign ions in the cerium lattice sites, and (3) concentration of oxygen vacancies.^14^ A detailed discussion regarding oxygen vacancies in ceria is provided by Ganduglia-Pirovano et al.^15^ The influence of dopants on the properties of ceria nanomaterials in thus-far studied systems can be found in an interesting review by Abdulwahab et al.^16^

Thus far, the most commonly studied doped ceria support in heterogeneous catalysis has been Zr-doped ceria.^17–19^ However, other dopants have received some attention, such as Cu-doped ceria, Mn-doped ceria, and Cr-doped ceria.^20–25^ These have been tested in exhaust cleanup, especially CO oxidation and NO elimination.^21,22^ In the study of Cu-doped ceria, it was found that the copper is present both in the ceria lattice and as a separate phase on the surface.^20^ An interesting study on the doping of CeO_2_ with Al^3+^ ions indicates that the introduction of these ions into the ceria lattice can “increase oxygen vacancy concentrations, enhance surface reactivity, and improve the reducibility of the catalysts”.^26^ Another study with Al-doped CeO_2_ revealed that the doping can substantially impact the acid/base properties of the oxide surface, such as pyridine adsorption.^27^ One of the studies on Mn-doped ceria revealed a solubility limit of manganese ions in the ceria lattice.^24^ The limits of dissolution of transition metals in CeO_2_ is the topic investigated in a paper by Ducka et al.^28^

In the context of the above-mentioned studies and lack of comparable experiments with different dopants in the ceria lattice, the main focus of the current study was the investigation of the influence of zirconium, aluminum, strontium, and magnesium ions on the interaction of the support with silver and the activity of the obtained catalysts in soot combustion. In order to enable a meaningful comparison between the systems, some restrictions were made in the choice of the type and quantity of dopant. Only monovalent dopants were chosen for this study, with two M^2+^ ions, namely, Sr^2+^ (a typical ceria dopant) and Mg^2+^, which substantially differ in their ionic radius: the latter exhibiting the same ionic radius as the commonly studied zirconium ion, the former being approximately twice as large. The dopant quantity was 5% (10% where possible) in order to avoid inducing changes in the ceria lattice parameter. Moreover, some simplifications in activity measurements were required. The main one being the performance of tests only in synthetic air, i.e., the stream lacked components such as NO_*x*_, H_2_O, and SO_2_, which are typically found in a realistic diesel exhaust stream and are often present in studies on soot combustion.^29–31^

Time-of-flight secondary ion mass spectrometry has been only sparingly applied in the study of catalysts despite the high lateral resolution and precision and the fact that it exhibits a lower detection limit and smaller probing depth than most other surface-sensitive techniques. It has been shown that ToF SIMS can be implemented in determining critical information about catalyst surfaces for Fischer–Tropsch synthesis,^32^ ammonia synthesis,^33^ as well as catalytic systems for other reactions.^34,35^ It is excellent at showing the presence of, e.g., a potassium layer on the surface of a catalyst precursor^32^ or the emission of KFe^+^ and KFeO^+^ ions from the studied surface, which indicates the possibility of formation of Fe–K bonds. A ToF-SIMS study on Co/SiO_2_ catalysts gave a lot of valuable information to the authors regarding their systems, which was not possible to attain by any other technique,^32^ such as the relative ratio of the components, i.e., cobalt and silicon, along with their distribution on the surface, as well as much more in-depth information, such as relative ratios of fragments/ions, e.g., Co^+^, CoH^+^, SiOCo^+^, and SiHO_2_CO^+^. Moreover, one ToF-SIMS study clearly indicated that an interaction between gold and the support in catalysts (Au/Al_2_O_3_ and Au/TiO_2_) leads to the emission of AuO^–^, AuOH^–^, and AuO_2_^–^ ions, which were not detected upon bombardment of a gold foil with primary ions.^36^ The importance of the presence of oxygen-containing ions detected in the ToF SIMS spectra emitted from a RuO_*x*_/Ce_0.85_Zr_0.15_O_2_ catalyst surface as well as from the support after silver deposition has been shown in our previous studies.^37,38^ However, otherwise, ToF SIMS has not been widely utilized to investigate interactions of the active phase and the support itself. This work aims to fill that gap.

## Experimental Section

### Catalyst Synthesis

Four solid solution supports were prepared for this study, each using 100 g of ammonium ceric nitrate (reagent grade, POCh Gliwice), which was dissolved in approximately 400 mL of redistilled water. Then, 0.0096 mol of one of the following compounds were dissolved in 50 mL of redistilled water: i.e., 2.57 g of zirconyl nitrate (reagent grade, POCh Gliwice), 3.60 g of aluminum nitrate (reagent grade, POCh Gliwice), 2.03 g of strontium nitrate (reagent grade, POCh Gliwice), and 2.46 g of magnesium nitrate (reagent grade, POCh Gliwice) and mixed with the other solution. The precipitation was performed with 100 mL of an aqueous ammonia solution, 15% (purum p.a. POCh, Gliwice, Poland) was added while stirring vigorously. The obtained solids were transferred into evaporating dishes and dried for 2 h at 100 °C and then heated to 550 °C in a muffle furnace with a heating ramp of 15 °C·min^–1^ and calcined for 4 h. Approx. 50 g of each support was impregnated with a solution made from 4.14 g of silver nitrate (reagent grade, POCh Gliwice) dissolved in 50 mL of redistilled water via wet impregnation. The catalysts were dried at 100 °C for 2 h and then calcined at 550 °C for 1 h. The undoped ceria was prepared in an analogous way from the same precursor using aqueous ammonia as the precipitating agent and the same preparation procedure.

### Catalytic Activity

All activity measurements were carried out using a Simultaneous Thermal Analysis instrument (model 449C) from Netzsch in a flowing stream (90 mL·min^–1^) of synthetic air (21% O_2_, 79% N_2_, Multax) with a temperature ramp of 10 °C·min^–1^. Tight contact of soot was ensured by grinding 0.10 g of support or catalyst with 0.02 g of the model soot (low ash content, <2%, active carbon from Merck) using a mortar and pestle for 60 s. Each DTA-TGA run was performed with approximately 25 mg of the sample. The weight percent is related to the entire mass of the sample. Measurements were also conducted in tight contact with extended grinding (5 min) and in loose contact, which was achieved through shaking the mixture of soot and catalyst for 1 min in a closed Eppendorf vial. Additional measurements with carbon black (Super P, IMERYS) were performed for comparison. Moreover, measurements with heating rates of 5 and 15 °C·min^–1^ were performed for selected catalysts to enable *E*_a_ determination.

Isothermal measurements were performed in tight contact in the TGA-MS and TGA modes to allow for a larger sample size by using a large, flat pan. The entire experiment was carried out in a flowing stream of synthetic air with a heating ramp of 10 °C·min^–1^ in each heating segment. There were two thermal programs for the isothermal experiments. In the first, the sample was heated to 420 °C, held at this temperature for 3 h, heated to 440 °C, held at 440 °C for 3 h, heated to 460 °C, held at 460 °C for 3 h, heated to 480 °C, and held at 480 °C for 3 h. In the other, the sample was heated to a specific temperature (420 °C, 440 and 460 °C) and held at that temperature for 9 h.

The cyclic tests were also carried out in tight contact in TGA mode. In each cycle, the temperature was increased to 850 °C at a rate of 10 °C·min^–1^, after which the catalyst was cooled, mixed with a new portion of soot, and heated to 850 °C (10 °C·min^–1^).

### Catalyst Characterization

The samples were tested using numerous characterization techniques, including:

X-ray diffraction (XRD) studies were performed with a D5000 Bruker AXS instrument, equipped with a LynxEye detector, operating at 1.5418 Å (Cu Kα radiation), 40 kV, 40 mA in the scattering angle range of 20–140°. The data was used to calculate the particle size and lattice strain. The lattice constants of the supports were obtained using the Nelson-Riley function based on the positions of all measured reflexes.

#### Scanning Electron Microscopy–Energy-Dispersive X-ray Spectroscopy

The supports and catalysts were imaged with a Prisma E instrument (ThermoScientific). The Everhart– Thornley Detector (ETD) and Circular Backscatter Detector (CBS) were used to simultaneously image the same particles at a working distance of 10 mm, spot size of 4, voltage of 15 kV, and magnifications between 2000 and 20,000 times. The elemental maps were acquired with a spot size of 6.

#### Time-of-Flight Secondary Ion Mass Spectrometry

The ToF SIMS detector was mounted onto a Helios 5 microscope (Thermo Fisher Scientific) equipped with several detectors, including the Everhart-Thornley Detector. The measurements were made using a Xe focused ion beam with a beam voltage of 30 kV and a beam current of 15 nA.

#### Nitrogen Physisorption

The specific surface area (*S*_BET_) as well as pore type, size, and volume were determined using an ASAP 2020 (Micromeritics) instrument. Prior to measurements, the sample (1 g) was degassed in the following manner: the evacuation was conducted at a rate of 5.0 mmHg·s^–1^ to 500 μmHg, held for 60 min, then the sample was heated to 150 °C with a 10 °C·min^–1^ ramp and the temperature was held for 240 min. Following the cool-down and backfilling, the experiments were conducted at −169 °C using nitrogen pulses in the *p*/*p*_0_ range of 0.01–1.0. The specific surface area was obtained by using the Brunauer–Emmett–Teller model. The Barrett–Joyner–Halenda (BJH) model was used for the assessment of pore size and pore volume.

#### X-ray Photoelectron Spectroscopy (XPS)

The measurements were used to determine the surface composition and chemical state of elements in the samples (KAlpha instrument, Thermo Scientific). Detailed scans of C 1s, O 1s, Ce 3d, Ag 3d, and those of the dopants were acquired. Peak fitting was performed when a Shirley-type background was subtracted, and all signals were shifted to align C 1s to 284.8 eV. The fitting of the Ce 3d region was performed with 10 components to allow for the Ce^3+^/Ce^4+^ ratio determination.

#### Energy-Dispersive X-ray Fluorescence (EDXRF)

The measurements were carried out using MiniPal 4 equipment from PANalytical Co, with a Rh-tube and silicon drift detector (resolution of 145 eV). The spectra were collected in ambient air atmosphere, without the use of a filter, at a tube voltage of 30 kV. The time of acquisition was set to 600 s, and the tube current was up to 81 μA.

#### Raman Spectroscopy

The measurements were carried out with a Nicolet Almega Dispersive Spectrometer (Thermo Fisher). The laser wavelength was 532 nm with 65% laser power. The following parameters were used: aperture 100 μm pinhole, high resolution (3.2–4.8 cm^–1^), spot size 1.1 μm, multiple grating positions, wavenumber range 100–3500 cm^–1^. The data was analyzed with Omnic software (ThermoFisher).

#### Pyridine Desorption Fourier Transform Infrared Spectroscopy

The studies were performed on a Nicolet 6700 spectroscope (ThermoFisher). The following parameters were applied in the experiments: CaF_2_ windows, 32 scans, wavenumber range 1300–4000 cm^–1^. The spectra were acquired after the samples were degassed for 4 h at *T* = 550 °C, *p* = 4·10^–1^ hPa, and exposed to pyridine for 5 min. The pyridine desorption took place for 10 min at RT and temperatures between 150 and 350 °C with 50 °C step. The data was analyzed with Omnic software (ThermoFisher).

Hammett indicator tests were performed with chalcone, dibenzylideneacetone, crystal violet, methyl red, bromothymol blue, phenolphthalein, 2,4-dinitroaniline, 4-nitroaniline, diphenylamine, 4-chloroaniline, and triphenylmethane. The tests were conducted as follows: (1) the samples were heated to 300 °C for 2 h, (2) approximately 30 mg of each sample was put into a glass vial, (3) four drops of the 0.1 wt % toluene solution of an indicator were poured onto the support, and (4) the colors of the support were recorded after 24 h.

#### Temperature-Programmed Reduction (TPR)

The measurements were performed using an Autochem II 2920 (Micromeritics). The procedure consisted of (1) 5 min rinsing with a 10%H_2_/Ar gas mixture at 30 mL·min^–1^ with simultaneous baseline signal stabilization, then (2) heating to 850 °C at a rate of 10 °C·min^–1^ in a flowing 10%H_2_/Ar mixture at 30 mL·min^–1^. Changes in hydrogen concentration in the outlet stream were recorded using a Thermal Conductivity Detector (TCD). Prior to the experiments, a five-point calibration curve of the response of the TCD was obtained.

## Results and Discussion

The ionic radii of Ce^4+^ and Ce^3+^ are approximately 0.97 and 1.14 Å, respectively, whereas those of the dopants, namely, Zr^4+^, Sr^2+^, Al^3+^, and Mg^2+^, are 0.84, 1.18, 0.54, and 0.82 Å, respectively. The diffraction patterns of ceria doped with these ions can be seen in Figure 1A. They contain reflexes arising only from the fluorite-type phase, which is typical for undoped ceria (PDF#34–0394), including (111), (200), (220), (311), etc. The lattice constants of the supports are 0.541 ± 0.001 nm for all of the studied systems. In an investigation on the influence of rare earth metal ion doping on the ceria lattice constant, a linear dependence was noted on the radius of the dopant cation ranging from 0.539 nm for Yb^3+^-doped ceria to 0.545 nm for Nd^3+^-doped ceria.^39^ Eguchi et al. reported that when ceria is doped with strontium, its lattice constant increases from approximately 0.541 nm to slightly more than 0.543 nm at the solubility limit, which has clearly not been reached in our study. In contrast, for magnesium as a dopant, no change of the lattice parameter was noted for the studied range of Mg^2+^ concentrations, and was consistently approximately 0.541 nm,^39^ which is the same as the value obtained in our study (Table 1). It is beneficial to conduct a study with supports that do not differ in the lattice constant because then the measurements allow a meaningful comparison between the results: the crystal lattice is the same and only the dopant is different (Table 1). It is noteworthy that the width of the peaks of ceria in this study substantially differs and strongly depends on the type of dopant used (Figure 1), but it does not correlate with the ion size. This indicates that the introduction of a small amount of the dopant influences the crystallite size. The narrowest peaks were noted for the Sr^2+^-doped sample. This corresponds to particles with an average size of 12.1 nm. The particle sizes are listed in Table 1. The average particle size of the Mg^2+^-doped sample is 8.9 nm, and that of Zr5Ce is 7.9 nm. The only value that was substantially different was the one calculated for the catalyst on the Al^3+^-doped sample, which had the widest peaks (Figure 1A), i.e., 6.3 nm.

**Figure 1:**
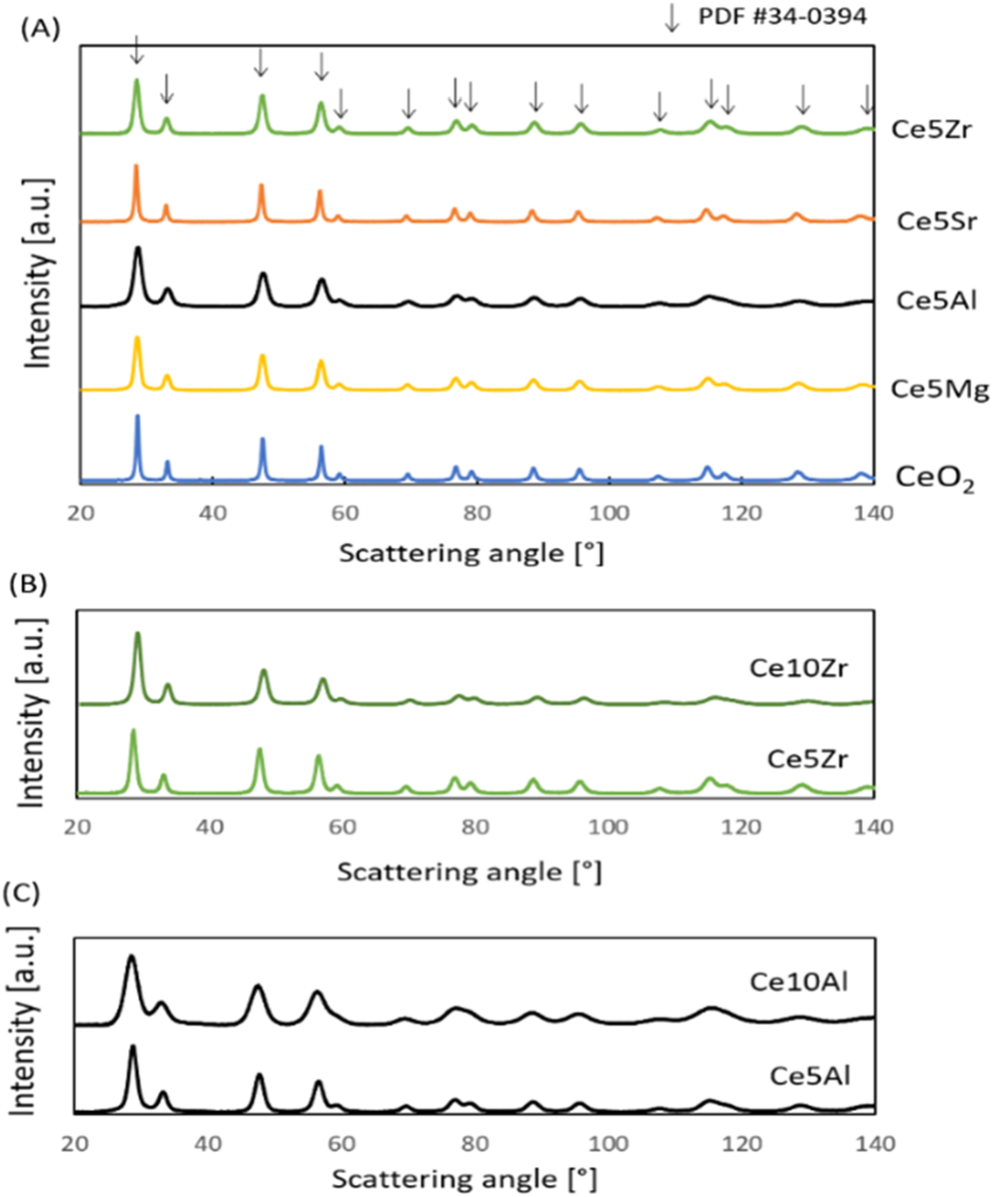
X-ray diffraction patterns of (A) the solid solutions with 5 mol % of dopant and comparison of 5 mol % and 10 mol % of (B) Zr^4+^ and (C) Al^3+^ ions.

**Table 1:** XRD Results of Silver Catalysts Supported on Solid Solutions Containing 5% of the Dopant

		Ag catalyst supported on ceria doped with		
parameter		Zr^4+^	Sr^2+^/Mg^2+^	Al^3+^
lattice constant	*a*_supp_ [nm]	0.540	0.542/0.541	0.541
	*a*_Ag_ [nm]	0.409	0.409/0.409	0.409
crystallite size	*D*_supp_ [nm]	7.9 ± 1	12.1/8.9 ± 1	6.3 ± 1
	*D*_Ag_ [nm]	78 ± 5	76/82 ± 5	80 ± 5
steric strain	ε_supp_	0.004	0.006/0.004	0.005
	ε_Ag_	0.002	0.002/0.001	0.001

In order to gain further insight into the influence of the dopants on the crystallite size, two more supports were synthesized: one with 10% zirconium ions and the other with 10% aluminum ions. The diffraction patterns are compiled in Figure 1B,1C, respectively. When one considers the example of supports doped with 5 and 10% Zr^4+^, two changes can be observed: (1) the increase of the concentration of Zr^4+^ leads to only a slight increase of the peak breadth, which means that the size of particles is only slightly smaller (*D* = 7.0 nm), but (2) there is a visible shift of the peaks to higher 2-θ values, which means that the lattice parameter of ceria has decreased (0.538 nm). It is commonly known that the incorporation of zirconium ions into the ceria lattice leads to a decrease in the lattice constant of ceria.^31,40^ However, when the content of Al^3+^ is increased to 10%, the peaks were visibly broader as illustrated in Figure 1C, which translates to a substantially smaller crystallite size (2.8 nm), but there is no change in the position of the peaks in the diffraction pattern despite the fact that Al^3+^ ions exhibit a smaller ionic radius than Zr^4+^. The aluminum ion content has a much more pronounced effect on the crystallite size than any other dopants studied. The influence of the presence of a dopant on crystallite growth has been explored by Pijolat et al.^41^ The authors stated that there are different reasons for the way in which guest ions impact the crystalline growth of the solid solution. In the case of Mg^2+^, the effect is attributed to its “strong tendency to form associates with oxygen vacancies”,^41^ whereas for Al^3+^ ions, the reason provided for its influence is “a strong tendency to form associates with Ce^3+^ ions”.^41^

The diffraction patterns of the catalysts are compiled in Figure 2. There are only two phases present in the catalysts: a solid solution support and metallic silver (PDF#040783). The lattice constant of metallic silver is 0.409 nm on all of the supports with very little steric strain of the silver lattice (Table 1). The fact that with ceria supports silver is only present in one specific form, i.e., metallic silver, as opposed to different forms of silver found on other oxides, has already been reported by Sawatmongkhon et al.^42^ The values in Table 1 indicate that the main difference between the silver deposited onto the different solid solutions is the particle size. The silver particles are the smallest, 65 nm, for the Al^3+^-doped sample and the largest for Mg^2+^-doped ceria, 82 nm (Table 1). Perhaps this is also due to the formation of associates with different components of the ceria structure. The number of phases associated with each component determined using XRD analysis and the type of structures in which they are present are compiled in Table S1 (Supporting Information).

**Figure 2:**
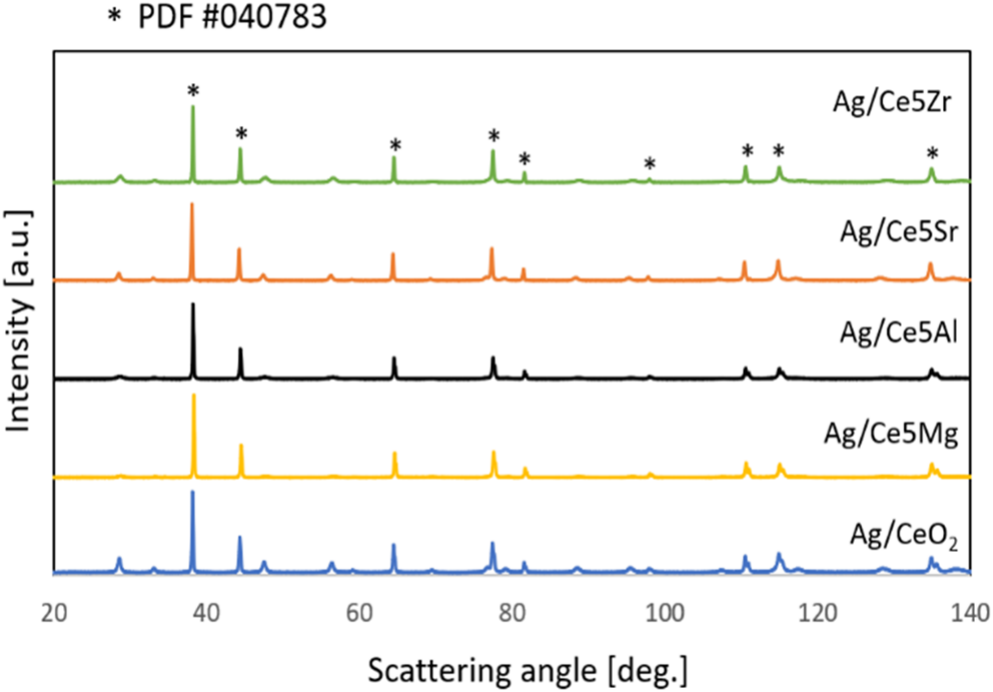
X-ray diffraction patterns of the silver catalysts.

The introduction of dopants into the ceria lattice influences the porosity of the obtained silver catalysts. Both the types of pores and their amounts were impacted. The hysteresis loops are type H1 for Ag on Zr^4+^-doped ceria (Figure 3A). This means that in the case of Ag/Ce5Zr, the pores are narrow and open at both ends.^43,44^ The incorporation of strontium ions into the ceria lattice during the synthesis leads to the formation of silver catalysts with slit pores (micropores) and hence an H4 type hysteresis loop for Ag on Sr^2+^-doped ceria (Figure 3B). An H2a-type loop was recorded for Ag on Al^3+^-doped ceria (Figure 3C), which indicates the presence of wide body pores or ink bottle-like pores in the catalyst supported on Al^3+^-doped ceria. It is noteworthy that the hysteresis loop recorded for the Mg^2+^-doped ceria is similar to H1-, H4-, and H2b-type loops (Figure 3D). These types of pore types are very closely related, and all three can be seen in doped ceria solid oxide solutions.^45,46^ It is particularly interesting that zirconium-doped ceria has been shown to exhibit all three types of pores,^47,48^ which indicates that there are many parameters during their synthesis that influence the resultant pore shape. The study by Li et al.^47^ shows that the synthesis method can change the pore type of such oxides. In fact, the same is true for undoped ceria,^49–51^ i.e., different synthesis routes lead to differently shaped pores. It can be seen that the Ce10Al support itself has the same type of pore shape as the resulting catalyst (Figure 3E), but in a larger quantity. The shape of the hysteresis loop is different from that observed for Ce5Al, which shows that the concentration of aluminum ions in the ceria lattice affects the shape of the pores. Considering the fact that the particles are smaller, as evidenced by the XRD results, this is not entirely unexpected. Moreover, this is in line with the fact that the surface area of Ag/Ce10Al, i.e., 33 m^2^/g, is also higher than that noted for Ag/Ce5Al (Table 2).

**Figure 3:**
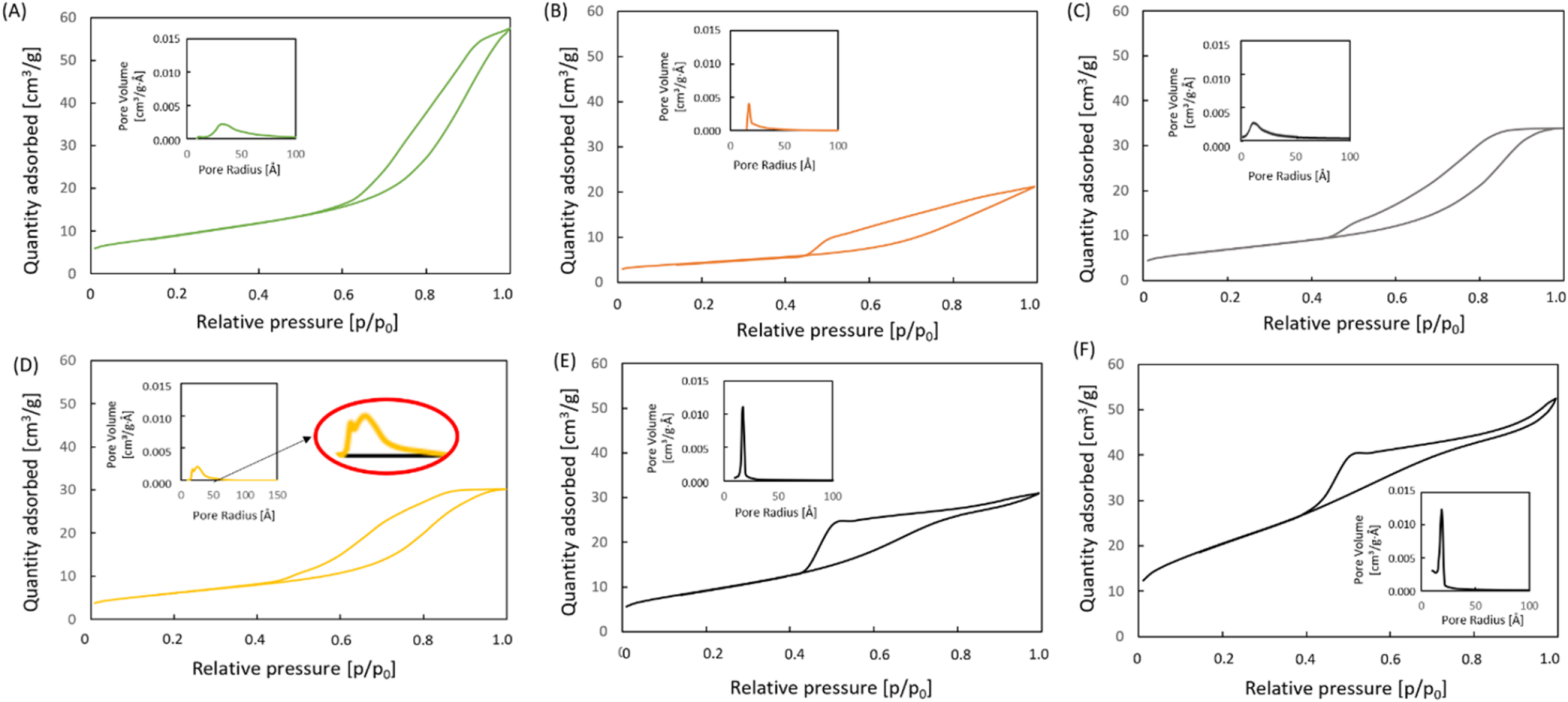
Nitrogen physisorption results obtained for: (A) Ce5Zr, (B) Ce5Sr, (C) Ce5Al, (D) Ag/Ce5Al, (E) Ag/Ce10Al, and (F) Ce10Al.

**Table 2:** Results of Characterization Studies of Silver Catalysts Supported on Solid Solutions Containing 5% of the Dopant

		Ag catalyst supported on ceria doped with		
parameter		Zr^4+^	Sr^2+^/Mg^2+^	Al^3+^
*S*_BET_	[m^2^/g]	31.9	15.3/21.7	24.3
*V*_P_	cm^3^/g	0.090	0.033/0.047	0.052
*r*_P_	2 V/A [Å]	48	39/37	46
XRF	dopant [wt %]	4.1^a^	0.9/0.8^a^	3.8^a^
		3.5^b^	0.4/0.21^b^	1.9^b^
	Ag [wt %]	38.1	52.5/64.6	37.6
XPS	dopant [wt %]	5.5^a^	0.5/0.7^a^	4.0^a^
		0.5^b^	0.1/0.1^b^	1.5^b^
	Ag [wt %]	89.8	91.5/90.6	87.5

a Support.

b Catalyst.

The different dopants clearly influence the pore radius in the produced supports. This can be seen in the insets in Figure 3. All of the *y*-axes have been unified to allow a comparison of the shape and magnitude of the pore volume as a function of the pore radius of the studied catalysts. The Zr^4+^-doped catalyst exhibits a broad peak at around 30 Å, whereas both catalysts supported on Sr^2+^-doped ceria and Al^3+^-doped ceria have a very narrow maximum at 17 Å. The catalyst supported on Mg^2+^-doped ceria has two local maxima: one at 17 and the other at 30 Å. A similar bimodal distribution of pore sizes, along with a detailed discussion regarding pore distribution of ceria depending on how it is synthesized, can be found in a paper by Sakina et al.^52^ In our study, however, the synthesis method was the same, but the variable that was changed was the dopant used in the preparation of the ceria. These results show that the dopant could be used to control this parameter.

Elemental maps of all of the samples were acquired using SEM-EDX. Those of the solid oxide solutions show a uniform distribution of the dopant within the host oxide. An example of the zirconium-doped ceria is provided in Figure 4A, and the maps of the other solid oxide solutions are shown in Figure S1 (Supporting Information). The EDX analysis does not allow for identification of the structure/phase in which a given element is present, but the simultaneous increase/decrease of the relative abundance of elements in the same spots indicates that they are components of a common phase. This can be seen in the elemental maps of the physical mixture of the two oxides, which reveal the presence of two separate oxides in different parts of the image (Figure 4B). The spots in which the zirconium intensity is increased (bright spot) are accompanied by the loss of intensity of cerium (darker spot) and vice versa, whereas oxygen is present in the spots with high intensity of both cerium and zirconium. This means that both zirconium and cerium are present with oxygen. The same is also true for the other physical mixtures (Figure S2, Supporting Information). The number of phases as seen in the elemental maps and circular backscatter images, which differentiate phases on the basis of elemental weight, is compiled in Table S1 (Supporting Information). In such an analysis, it has to be taken into account that hydrogen is not detected by EDX and adventitious carbon is present on all surfaces, which may hinder the identification of all elements present in a compound.

**Figure 4:**
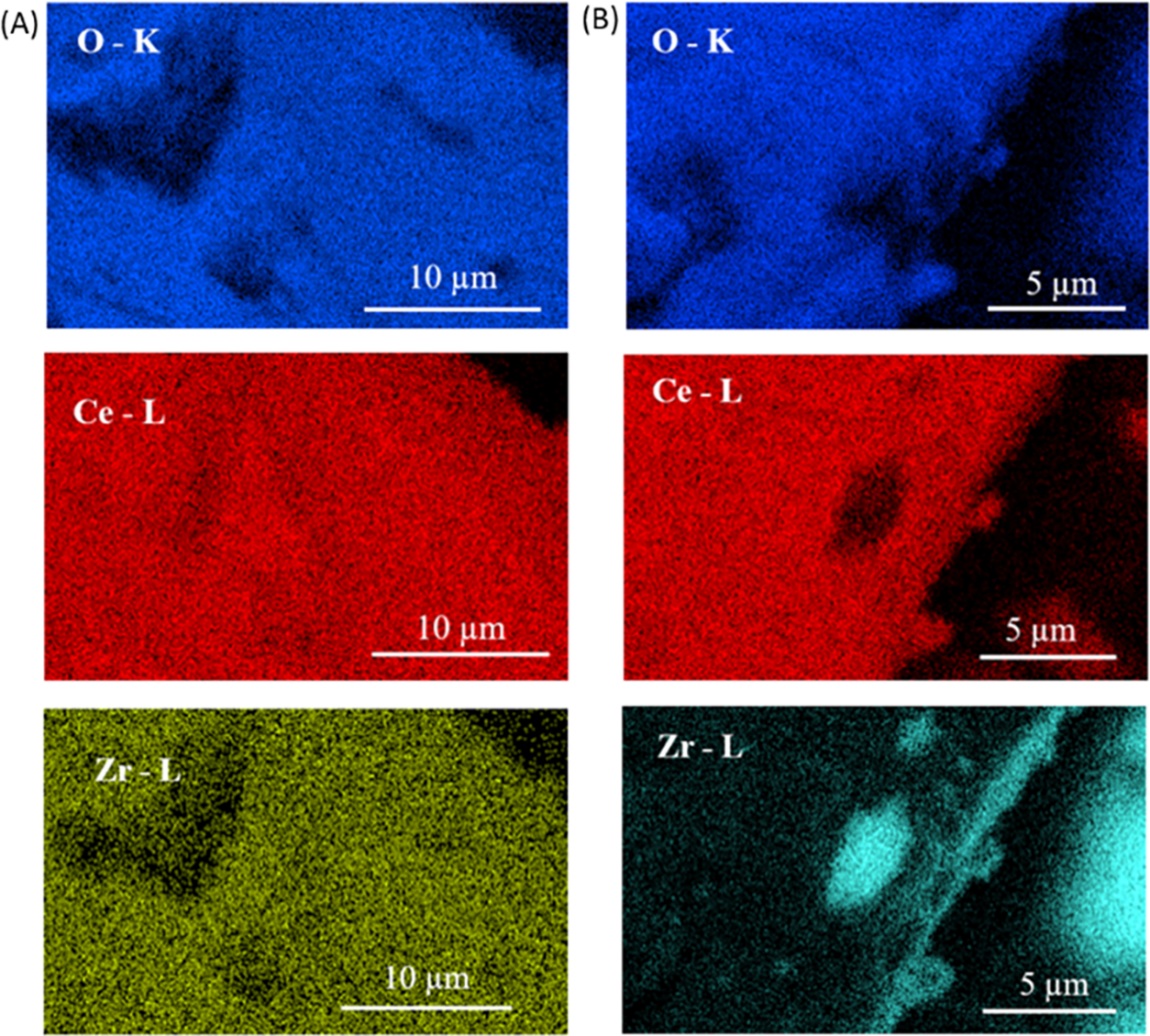
Energy-dispersive X-ray spectroscopy results obtained for: (A) Ce5Zr and (B) a physical mixture of CeO_2_ and ZrO_2_ (Ce:Zr = 95:5).

The silver catalysts obtained using the solid solution supports all show a similar property, namely, that the entire surface is coated with silver. Two examples of SEM images and the elemental maps of Ag, O, and Ce, namely that of Ag/Ce5Zr and Ag/Ce5Al, are seen in Figure 5. The elemental silver maps exhibit a layer of silver on the surface. The darker parts of that image indicate areas in which there is relatively less silver, with simultaneous intensive coloring in the elemental maps of cerium and oxygen. This indicates that cerium can only be seen in the pieces of the support that fell onto the catalyst after deposition or in spots where the silver layer was peeled off. The same was observed for the catalysts produced using the other solid solutions, namely Ag/Ce5Sr and Ag/Ce5Mg (Figure S3).

**Figure 5:**
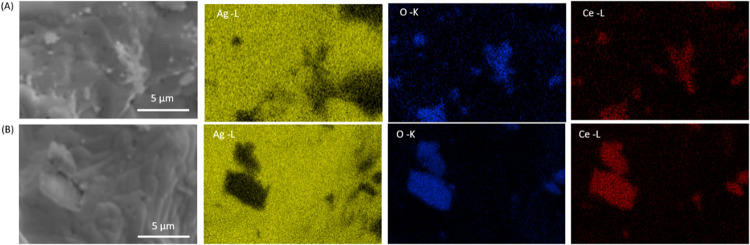
Energy-dispersive X-ray spectroscopy results obtained for the catalysts: (A) Ag/Ce5Zr and (B) Ag/Ce5Al.

X-ray Photoelectron Spectroscopy was applied for examining both the supports and catalysts. The survey spectra are used to show the elemental composition of the surface. The spectra obtained for the Zr-doped and Al-doped ceria and catalysts are illustrated in Figure 6. They show that the only elements found on the surface of these are Ce, O, Zr, or Al, C, and, in the case of the catalysts, also Ag. This is not the case for the Sr-doped ceria- and Mg-doped ceria supported catalysts, for which the dopant is not detected with XPS under the layer of silver. The fact that the silver is present in the form of metallic silver is indicated by the loss features seen in the Ag 3d detailed region spectra (shown in the spectrum insert, Figure 6). This is also true for the other catalysts. The oxidation states of silver as determined by XPS are compiled in Table S1 (Supporting Information). The fact that it forms a layer that covers the surface of the supports can be seen in the domination of the XPS survey spectrum by this element and the substantial loss of intensity of both cerium 3d peak intensity and that of the dopant (Zr 3d region and Al 2p region, Figure 6C,6D, respectively). This can be seen for all of the studied catalysts. A compilation of the survey spectra of Ce5Al, Ce5Sr, and Ce5Zr as well as the silver catalysts obtained using these supports is shown in Figure S4 (Supporting Information). The supports doped with strontium and magnesium show a very low intensity of the peaks in the XPS spectra, whereas those in aluminum- and zirconium-doped ceria are consistent with Al^3+^ and Zr^4+^ ions in an oxidized state. The former shows an additional shoulder, which may indicate a different chemical environment, as in the case of doped alumina in our previous study.^53^ The fitting of the XPS data to investigate the Ce^3+^/Ce^4+^ ratio was performed with the standard 10 peak fitting, which shows a slight increase of this ratio upon doping (Table S1, Supporting Information). This is in line with reports found in the literature.^23,26,54^ Slightly higher values were obtained by Liu et al.^55^ The greatest increase was noted for the aluminum-doped sample. This indicates that the insertion of foreign ions of lower valency and the formation of vacancies can also lead to changes in the Ce^3+^/Ce^4+^ ratio as previously indicated in other studies.^26,54^

**Figure 6:**
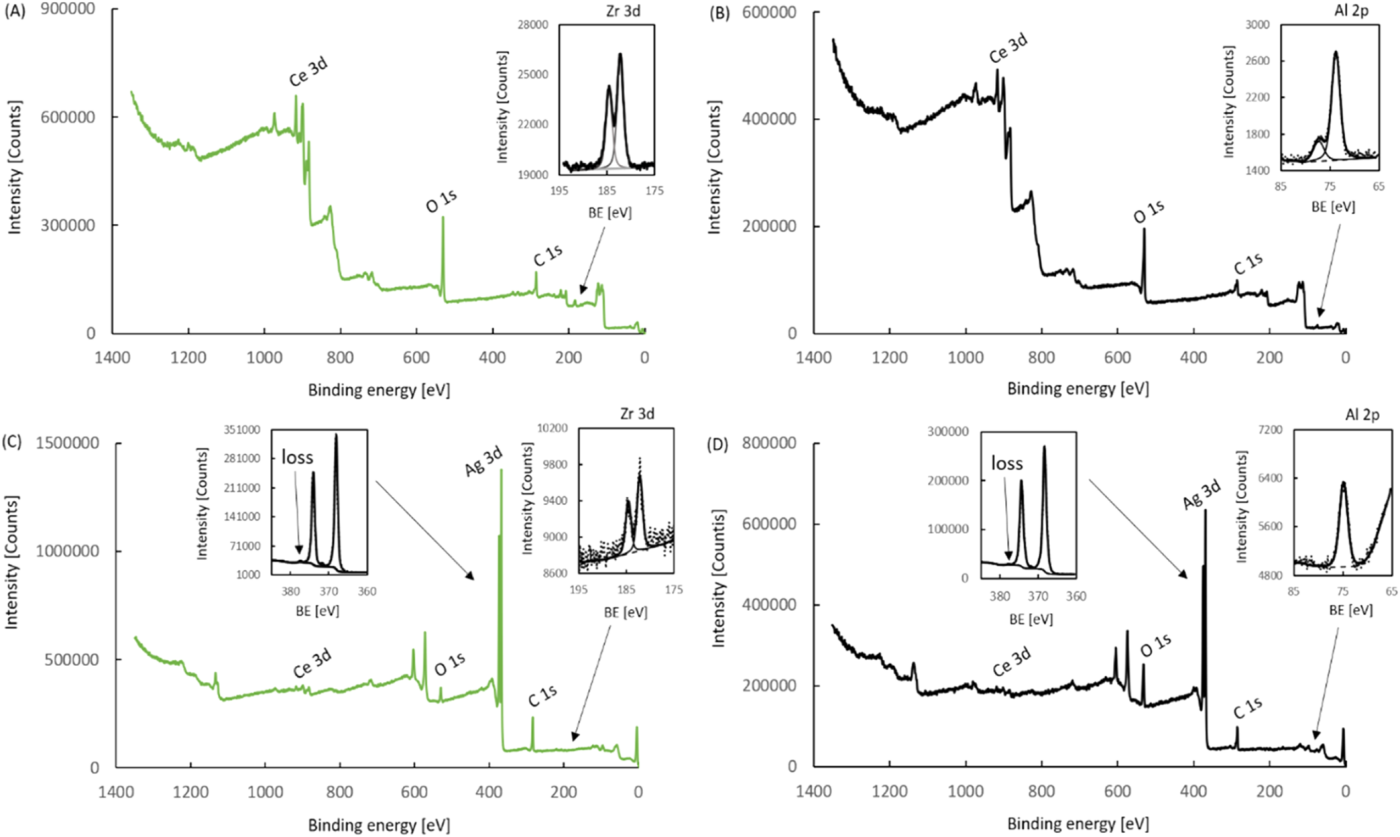
XPS survey spectra of supports (A) Ce5Zr and (B) Ce5Al, and the corresponding catalysts: (C) Ag/Ce5Zr and (D) Ag/Ce5Al.

The literature contains different active oxygen species considered to be involved in soot combustion using silver catalysts.^54–56^ Two possible options of the mechanism of soot oxidation proposed by Shimizu et al. are that either there are two reactive oxygen species that oxidize C to CO and then CO to CO_2_ or reactive molecular oxygen species that can oxidize C to CO_2_ in one step. In general, it is accepted that the active species are designated as O*_x_* ^*n*–^ or O*_n_* ^*x*–^ and that these species can form during the activation of O_2_ either on Ce^3+^ – *V*_o_ sites or through bulk-to-surface migration of oxygen within the ceria lattice. In both cases, the vacancies in the ceria lattice are important factors for the activity of Ag/CeO_2_ catalysts in soot combustion. The vacancies that form in ceria are sometimes divided into two types: the surface vacancies (*V*_O–s_) and the bulk vacancies (*V*_O–b_), and their relative ratio is crucial to the formation of the active species.^54,55^ There are different techniques that can be used to probe the oxygen vacancies. These include electron paramagnetic resonance (EPR) also known as electron spin resonance (ESR),^26,56,57^ as well as analysis of the detailed XPS Ce 3d region and Raman spectroscopy.^23,58–63^ Raman spectroscopy measurements have shown that each of the dopants influences the Raman spectrum in a different way (Figure S5, Supporting Information). Although each of the spectra has the same most intense band, i.e., the F_2g_ peak, its position and shape change depending on the dopant used. It is known from the literature that a red shift of this band and its broadening may be attributed to increased topological disorder and increased concentration of defects.^25,60^ The fact that the insertion of zirconium shifts the maximum of the main Raman peak by a few inverse centimeters to higher values has been shown in other studies.^59^ In the case of magnesium, no shift of the F_2g_ band was observed in our studies, though another study, which tested a wider range of Mg^2+^ concentrations, did find a shift of this feature.^61^ The broadening and shift of the F_2g_ band is the greatest for Ce5Al (Figure S5A). However, another feature, namely the band at approximately 600 cm^–1^, which is attributed to oxygen vacancies,^62,63^ is also substantially different for all of our studied solid solutions (Figure S5B,C). It is noteworthy that Ce5Al exhibits the least intensive signal, even compared to undoped ceria, whereas Ce5Zr, Ce5Sr, and Ce5Mg show a pronounced peak. The shift of this peak is the largest for the supports with the M^2+^ dopants, i.e., Ce5Mg and Ce5Sr.

The EDXRF method was used to gain information on the elemental composition of samples under investigation. The XRF spectra obtained for the catalysts supported on Ce5Zr and Ce5Sr are shown in Figure S6A,B, respectively (Supporting Information). They contain peaks from Ag, Ce, Zr/Sr, and Rh. Rhodium is present in all of the spectra due to the application of a Rh-tube in the instrument. The technique probes the entire sample, which allows detection of the dopant in all samples. The relative abundances of the components are provided in relative weight percent (Table 2). Since oxygen is not used in the calculations, the XPS values were also recalculated into wt % without oxygen (Table 2). The substantially higher silver content in the XPS experiments is a consequence of the fact that the silver is deposited onto the surface and XPS has a smaller probing depth than XRF. Moreover, the signals of the support, i.e., the cerium and the dopant, are strongly diminished (at the detection limit of the technique) in XPS spectra (Figure 6). It is interesting that in both techniques, the decrease in the cerium signal is much more pronounced than for the dopants. The results of the XRF measurement with ground particles of Ag/Ce5Zr show that the cerium content substantially increases, i.e., the Zr/Ce/Ag ratio changes from 3.5:58.4:38.1 to 2.8:77.1:20.1. This shows that the fact that silver forms a layer on top of the support affects these results.

The properties of the obtained solid oxide solutions were investigated using pyridine desorption Fourier Transform Infrared spectroscopy. The results are shown in Figure S7 (Supporting Information). The pyridine was adsorbed on the support wafers at room temperature for 5 min and then evacuated at RT for 10 min to ensure elimination of pyridine from the gas phase. Figure S7A shows that the entire *v*CCN frequency range (1700–1400 cm^–1^) is very similar for CeO_2_, Ce5Zr, and Ce5Al after pyridine adsorption. The overlays of the desorption curves recorded at RT and 150, 200, 250, and 300 °C are shown in Figure S7B–D, respectively. As found in the literature, ceria and zirconium-doped ceria do not exhibit the band associated with Brønsted sites (BPy).^64^ Neither does Ce5Al. Unlike in the case of pyridine desorption from the surface of low-temperature solid oxide solutions based on Al_2_O_3_,^53^ in the case of CeO_2_ and CeO_2_-based solid oxide solutions pyridine not only desorbs with a continuous decrease of the relative intensity of the band at 1440 cm^–1^, attributed to pyridine adsorbed on Lewis acid sites, i.e., LPy^64,65^ but with increasing desorption temperature gives a pronounced band that can be observed at 1466 cm^–1^ which arises from the oxidative breakdown of adsorbed pyridine.^27,70^ Although the undoped ceria (Figure S7B) and Ce5Zr (Figure S7C) behave similarly upon desorption of pyridine, a substantial difference was noted in the relative intensity of the latter band for Ce5Al (Figure S7D). It indicates that the doping of ceria with Al^3+^ ions leads to a hindrance of the oxidative breakdown of pyridine in comparison to undoped ceria and zirconium-doped ceria. In a study on chromium-, aluminum-, and sodium-modified CeO_2_, a similar finding was reported by Zaki et al.^65^ This was attributed to slowing the surface reactions, which contributed to the breakdown of pyridine. Hence, although Ce5Al has a higher Ce^3+^/Ce^4+^ ratio than the other supports, the change in the acid/base properties of the surface as a result of doping does not correspond to only the concentration of oxygen vacancies. These results indicate that factors other than the Ce^3+^/Ce^4+^ ratio alone contribute to the overall activity of the catalyst.

The influence of the dopant on the acid/base properties of the support was further tested by using Hammett indicators. The colors of the grains 24 h after the addition of the indicator solutions are given in Table S2 (Supporting Information). All of the grains were originally yellow, which in some instances made determination difficult, but clear differences have been observed, showing the impact of the type of dopant on the surface properties of the obtained low-temperature solid solutions. The aluminum-doped sample has the narrowest range of acidic sites. The colors noted for Ce5Zr and Ce5Mg show similarities of their acid/base properties, although similarities of Ce5Zr and Ce5Sr can also be found.

The results of the activity measurements of supports (green bars) and catalysts (orange bars) in soot combustion are listed in Figure 7. The lower the temperature of the maximum heat generation (*T*_max_), the higher the activity of the studied sample. The highest temperature was, therefore, noted for pure soot. The value noted for the undoped ceria was approximately 565 °C (Figure 7A). In the presence of the supports doped with 5 atom % of each studied host ion, a slightly lower *T*_max_ than that noted for undoped ceria was observed. Therefore, it can be concluded that the valence of the dopant is not a key factor that affects the activity of the support itself.

**Figure 7:**
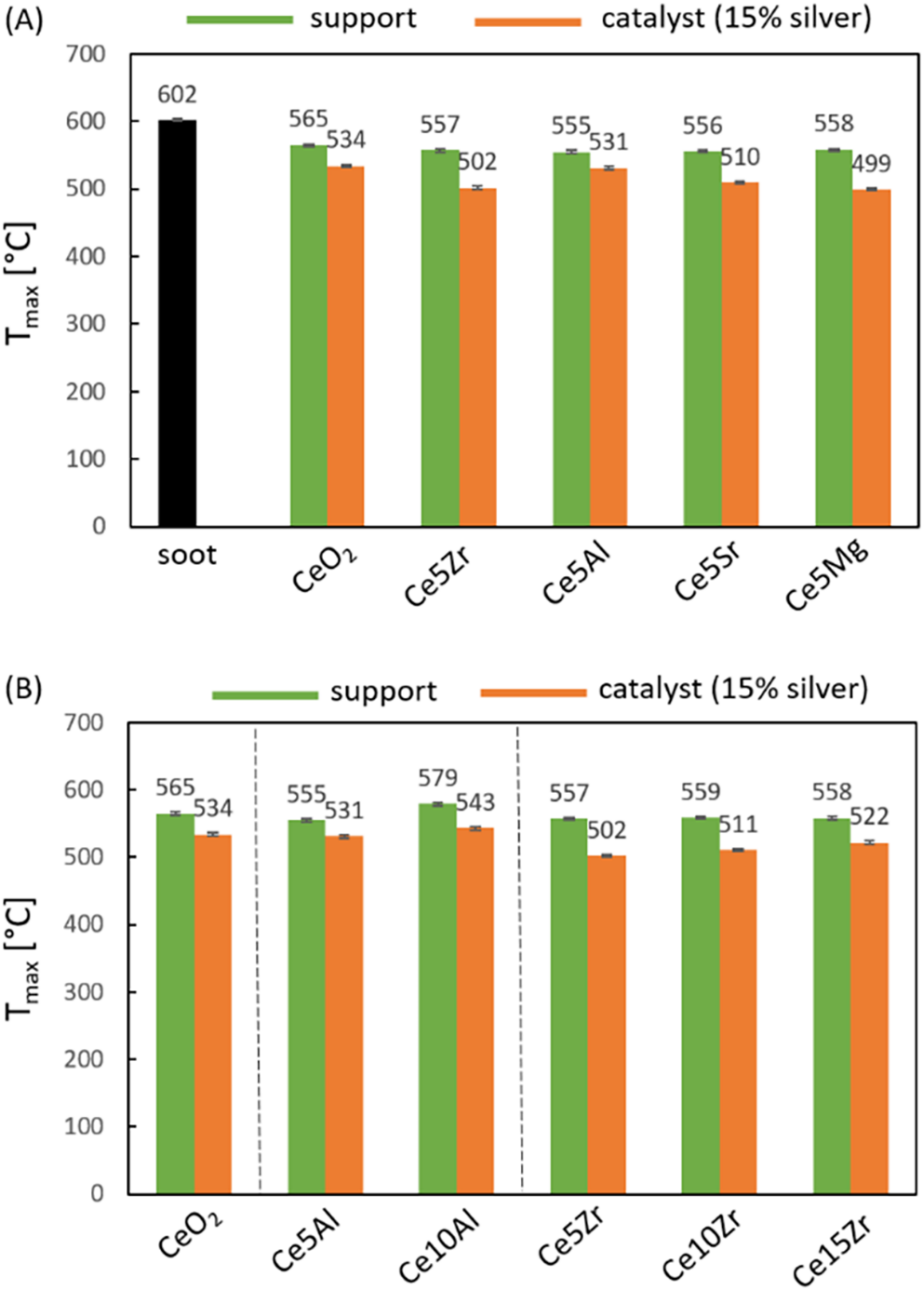
Temperature of the maximum heat generation of undoped ceria and ceria-type supports (green bars) and the obtained silver catalysts (orange bars); determined based on the maximum of the DTA signal for (A) catalysts and supports with 5% of dopant and (B) systems with different dopant loadings.

In order to check how the dopant concentration affects the activity of a support, an additional support with 10% Al^3+^ ions was synthesized. An increase in the concentration of the alumina ions in the ceria lattice from 5 to 10% yielded a noticeably less active support, with a *T*_max_ of 579 °C (Figure 7B, green bars), which is a higher temperature of the maximum heat generation than the *T*_max_ noted for the undoped ceria support. This, however, was not the case for zirconium-doped ceria. The results of activity studies of zirconium-doped ceria containing 5, 10, and 15 atom % Zr^4+^ ions are also compiled in Figure 7B. These results show that in the case of zirconium, no decrease of activity is noted upon the increase from 5 to 10 atom % Zr^4+^. A further increase (to 15 atom % Zr^4+^) does not lead to a decrease in the activity of the solid solution, either (Figure 7B). This reveals that the choice of dopant strongly influences the activity of the solid solution in soot combustion.

The deposition of 15 wt % silver onto the supports causes a substantial increase of the activity (Figure 7A,7B orange bars). The increase in activity upon silver deposition follows the order: Ag/Ce5Mg ≥ Ag/Ce5Zr > Ag/Ce5Sr ≫ Ag/Ce5Al. Nevertheless, the catalysts supported on the doped ceria supports are more active than those obtained with undoped ceria. The lowest improvement of activity was noted for the catalyst prepared with the Al-doped support, whose temperature of the maximum heat generation occurs at 531 °C (Figure 7A), which is very similar to that noted for the Ag/CeO_2_ system. Again, there is no clear dependence of the activity of the obtained system on the valency of the dopant.

When the aluminum ion content is increased to 10 atom %, the *T*_max_ in the presence of the catalyst is significantly higher (Figure 7B). Moreover, the increase in activity noted upon silver deposition onto this support is larger than that observed in the case of Ce5Al. Figure 7B also contains a compilation of activity results of zirconium-doped ceria supports and catalysts obtained on these oxides. The supports themselves exhibit the same activity in the studied range of dopant loading, which is different than what the studies showed for Al-doped ceria. However, although Ag/Ce5Zr, Ag/Ce10Zr, and Ag/Ce15Zr are all more active than undoped ceria, the gradual increase in the concentration of zirconium ions in the ceria lattice leads to a gradual decrease in the activity of the obtained catalyst in soot combustion. These studies reveal that for Al^3+^ and Zr^4+^, the smallest concentration (5 atom %) of a dopant leads to better catalytic supports than for higher dopant concentrations despite the fact that for all studied systems, there is only one phase, as evidenced by the XRD studies. In other words, although the addition of some dopant ions into the ceria lattice is beneficial, the insertion of more dopant ions has a detrimental effect on the overall activity of the silver catalysts.

Due to the common practice in soot combustion studies of performing measurements with loose contact or tight contact obtained by grinding the soot+catalyst mixture for a longer time (5 min or more)^55,66,67^ as well as the use of carbon black as a model soot,^66–68^ comparison experiments were conducted. Their results are compiled in Figure S8 (Supporting Information). Loose contact of soot and the catalysts yields substantially higher *T*_max_ temperatures than those noted for tight contact, which vary very little (Figure S8A). In fact, in experiments with loose contact, the 55° difference between Ag/Ce5Zr and Ce5Zr (Figure 7B) reduced to only 7° (Figure S8A). In contrast, the extension of grinding time to five min increases the differences between the *T*_max_ values of the catalysts (Figure S8B), but alters the shape of the DTA curve. The measurements with carbon black as the model soot were also performed with a 5 min-long grinding time. The results are presented in Figure S8C. First of all, there is a shift of the *T*_max_ of the heat generation of this model soot alone by almost 100 deg to a higher temperature in comparison to that of the active carbon. The trends remain unchanged, e.g., the catalyst obtained with the zirconium-doped support exhibits a higher activity than that synthesized with undoped ceria (Figure S8C).

**Figure 8:**
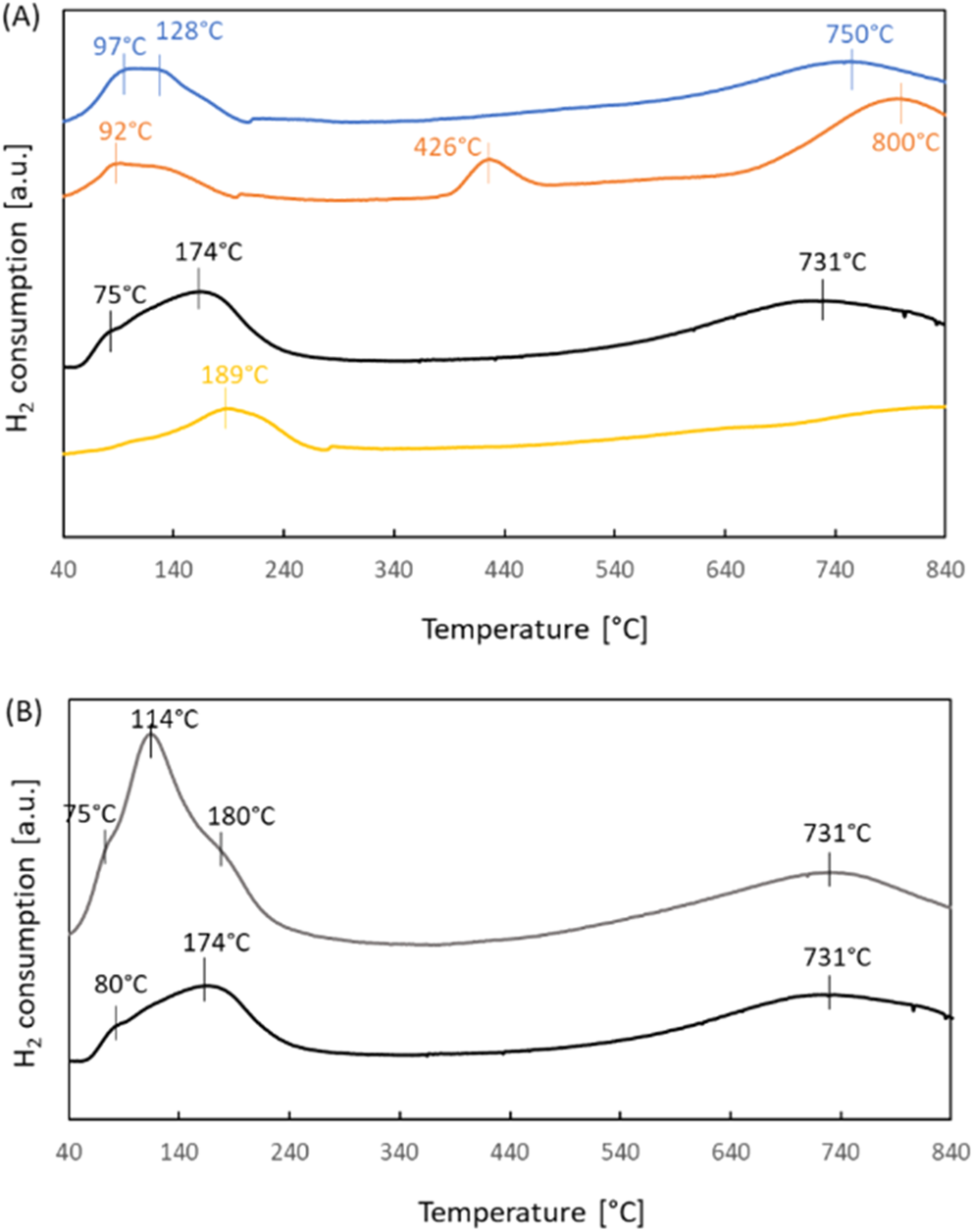
H_2_–temperature-programmed reduction curves of the catalysts: (A) Ag/Ce5Zr (blue), Ag/Ce5Sr (orange), Ag/Ce5Al (black), and Ag/Ce5Mg (yellow) and (B) Ag/Ce5Al (black) and Ag/Ce10Al (gray).

Additional soot combustion measurements with isothermal segments were performed to determine how the catalysts perform at lower temperatures during an extended time period. There were two types of isothermal experiments: one with increasing temperature (at 420 °C, 440 °C, 460 and 480 °C) separated by 3-h-long isothermal segments and the other with a single temperature isothermal hold for 9 h (at 420 or 440 °C). The results are compiled in Figure S9 (Supporting Information). In the first type of measurements, after being ground with soot for exactly one minute, Ag/Ce5Mg and Ag/Ce5Zr exhibited the highest activity in the catalytic tests, as seen in the enlarged image. Most of the combustion occurred within the first isothermal segment (Figure S9A). It can be seen that Ag/Ce5Sr shows a less steep mass loss than the other two. In contrast, Ag/Ce5Al shows a much slower rate of soot combustion, which is in line with the results of the activity tests presented in Figure 7A, which revealed that the Ag/Ce5Al system is the lowest-active system of those supported on a ceria solid solution. In the second type of measurements, a 5-min-long grinding time substantially improved the activity of catalysts. The curves obtained for Ag/Ce5Sr and Ag/Ce5Al at 420 °C are depicted in Figure S9B. Again, despite the improvement of activity, the performance of Ag/Ce5Sr is superior to that of Ag/Ce5Al. The increase in temperature from 420 to 440 °C (Figure S9) leads to a higher reaction rate of soot combustion, as evidenced by the change of the slope as illustrated with the curves recorded for Ag/Ce5Al. The activation energy of processes, such as soot combustion, is often determined using isoconversional methods,^56,69,70^ such as the Ozawa (or Ozawa-Flynn-Wall) plot or the Kissinger (or Kissinger-Akahira-Sunose) method, which require performing measurements at three different heating rates. Gross et al. used them to show that an increase in the calcination temperature of ceria leads to a change in the slope of the line, which was attributed to the easier formation of superoxide and peroxide species on the surface.^70^ Hence, additional measurements were performed with selected catalysts with ramps of 5 and 15 °C·min^–1^. The activation energies calculated based on the slopes of the *T*_10_ data in the Ozawa plots obtained with the active carbon as the model soot and 60 s-long grinding time are very high compared to those found in the literature, but it is noteworthy that the ratio of the *E*_a_ values noted for Ag/Ce5Zr and the least active catalyst (Ag/Ce10Al) equals 1.6, which indicates a substantial difference. As shown above, the elongation of the grinding time shifts the *T*_max_ to markedly lower temperatures (Figure S8, Supporting Information), e.g., for Ag/Ce5Zr, the *T*_10_ temperature is lower by 26° for a heating rate of 10 °C·min^–1^.

**Figure 9:**
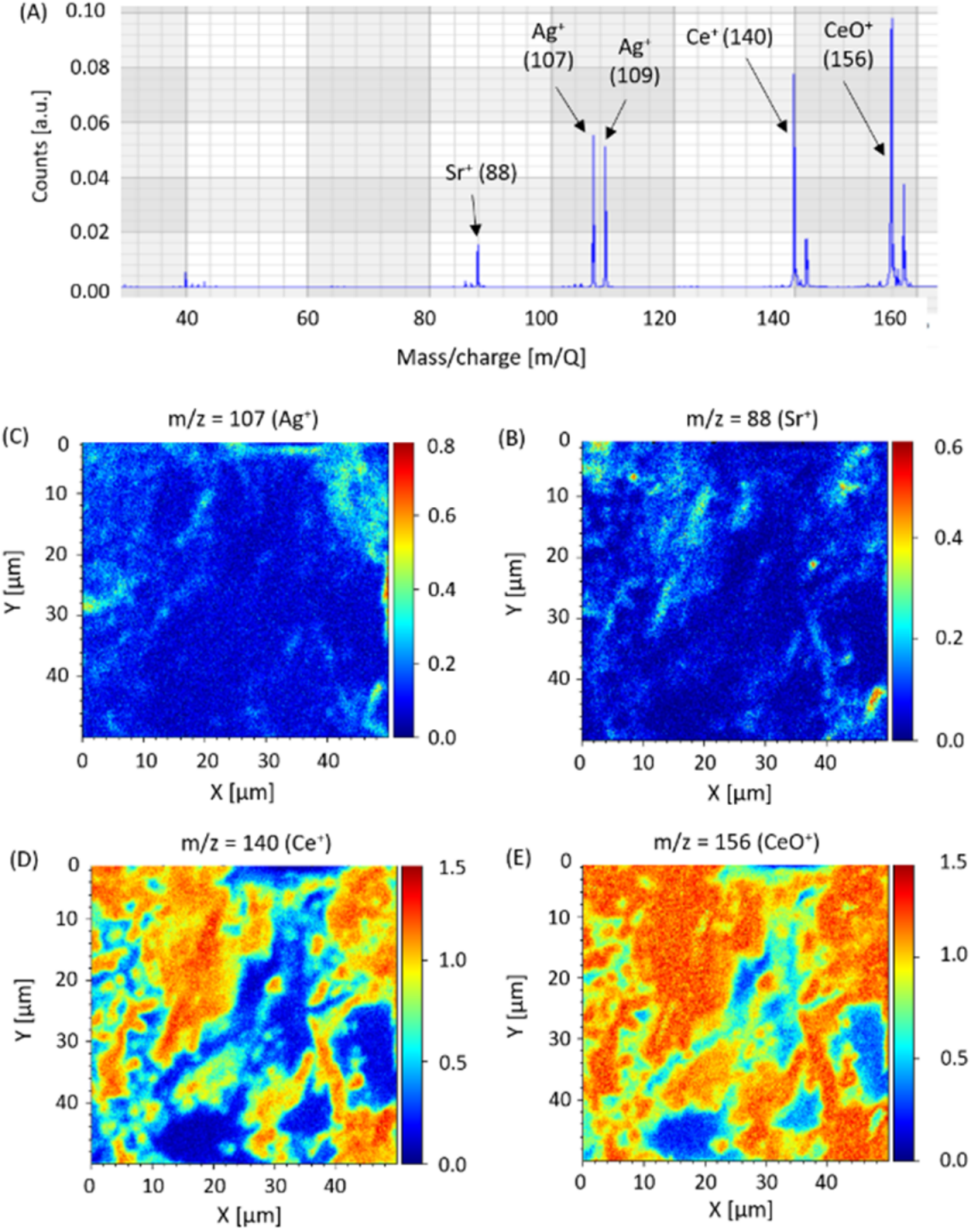
ToF SIMS positive ion spectrum of Ag/Ce5Sr: (A) the mass spectrum and ion distribution maps of (B) Sr^+^, (C) Ag^+^, (D) Ce^+^, and (E) CeO^+^.

Cyclic tests were also performed for Ag/Ce5Zr and Ag/Ce10Al, which revealed that in both cases, the activity in the first cycle was lower than in the second (Supporting Information, Figure S10). This effect was more pronounced in the case of the less active catalyst. The second and third cycles performed with Ag/Ce5Zr showed the same activity. The study of the morphology of the spent samples indicates that this might be due to the redistribution of silver from a flat layer to rounded particles formed on the layer (Figure S11, Supporting Information).

H_2_-TPR studies were used to determine the quantity of the oxidized silver species (low-temperature peaks) as well as the interaction of silver with the supports. The TPR curves obtained for the catalysts are substantially different, which shows how important the change in the dopant is for the reducibility of the catalyst (Figure 8A). The peaks below 240 °C are all attributed to the reduction of oxidized silver species. The order of the maximum reduction temperature shows that the Sr-doped and Zr-doped have very similar TPR curves, whereas the maximum reduction temperature is shifted to a higher temperature for both Al-doped and Mg-doped supports. The silver catalyst supported on the zirconium-doped ceria has a medium-sized low-temperature reduction peak with a plateau maximum between 97 and 128 °C. The catalyst supported on the strontium-doped ceria has a substantially smaller low-temperature reduction peak (2.5 cm^3^/g STP, Table 3) than the Zr^4+^-doped ceria-supported catalyst (3.9 cm^3^/g, Table 3). The largest concentration of oxidized silver species is found on the surface of the catalyst synthesized by using the aluminum-doped ceria (6.8 cm^3^/g, Table 3). A 2-fold increase in the concentration of Al^3+^ ions within the ceria lattice (Figure 8B) leads to a slightly lower than a 2-fold increase in this value (11.7 cm^3^/g, Table 3).

**Table 3:** Temperature-Programmed Desorption of CO_2_ Measurement Results of the Silver Catalysts Supported on Solid Solutions Containing 5% of the Dopant

	Ag catalyst supported on ceria doped with		
TRP peak	Zr^4+^	Sr^2+^/Mg^2+^	Al^3+^ (5/10)
*T*_MAX_ [°C]	97	92/189	174/114
H_2_ [cm^3^/g]	3.9	2.5/3.3	6.8/11.7
*T*_MAX_ [°C]	--	426/--	--
H_2_ [cm^3^/g]	--	1.2/--	--
*T*_MAX_ [°C]	750	800/>840	731/731

The data from the H_2_-TPR curves areas indicate that only the catalyst supported on Sr-doped ceria exhibits a peak in the midtemperature range, i.e., at 426 °C. The amount of hydrogen consumed in that reduction step is only 1.2 cm^3^/g, which is approximately half of the low-temperature hydrogen consumption. The temperatures of the high-temperature reduction maximum fall in the order: Ag/Ce5Al = Ag/Ce10Al > Ag/Ce5Zr ≫ Ag/Ce5Mg. The areas are difficult to quantify as none of the curves go back to the baseline before the end of the measurement. However, the largest rise is noted for the strontium-doped sample, and the least steep incline is observed for Ce5Mg, which again shows that the oxidation state of the dopant is not the main factor (if at all) contributing to the reducibility of the obtained catalysts. It is noteworthy that the increase in the concentration of Al^3+^ ions in the ceria lattice does not substantially impact the high-temperature reduction peak.

The ToF-SIMS spectra were collected in both the positive and negative ion modes. In both types of spectra, the most abundant ions are singly ionized, which means that the mass to charge ratio is mass/1 and the ions are 1+ or 1– despite their typical oxidation state. To exemplify this, a positive ion spectrum in the range of *m*/*z* = 30–160 of Ag/Ce5Sr is depicted in Figure 9A. Among the most intensive signals in that spectrum are *m*/*z* = 88, which is assigned to Sr^+^, *m*/*z* = 107 and *m*/*z* = 109, which are the two isotopes of silver, i.e., ^107^Ag^+^ and ^109^Ag^+^, *m*/*z* = 140, which was assigned to Ce^+^, and *m*/*z* = 156 (CeO^+^). In other words, despite the fact that strontium is present in the oxide as Sr^2+^ ions in the oxide and cerium was mostly Ce^4+^ or Ce^3+^, they show up in the positive ion spectrum as singly ionized cations. This can be illustrated by the lack of intensive signals at which these three would appear, namely, *m*/*z* = 44, *m*/*z* = 35, and *m*/*z* = 47, respectively (Figure 9A). The ion distribution maps of *m*/*z* = 88 (Figure 9B), *m*/*z* = 107 (Figure 9C), *m*/*z* = 140 (Figure 9D), and *m*/*z* = 156 (Figure 9E) show that the dopant and cerium cations (as well as CeO^+^) ions are more abundant in the same spots, which can be expected in true solid oxide solutions. The only map with a slightly different distribution is that of the Ag^+^ ions. It is noteworthy that strontium can be detected under silver, which was neither possible with EDX nor XPS.

The ToF SIMS studies indicate that the combination of the positive and negative ion spectra distribution maps provides much more insight into the interaction of the active phase, namely, silver, with the solid solution support. The ToF SIMS ion distribution maps for the positive and negative ions *m*/*z* = 27, *m*/*z* = 140, and *m*/*z* = 107 obtained from the same spot of Ag/Ce5Al are illustrated in Figure 10A,10B, respectively. The cations, i.e., Al^+^, Ce^+^, and Ag^+^, indicate that, as shown in Figure 9 for Ag/Ce5Sr, the distributions of the dopant and cerium look similar, whereas the distribution of silver is different. It appears as though the silver is distributed evenly on the surface. This, however, is different in the case of Ag ^–^, which closely follows the distribution of cerium and the dopant. When one looks only at the negative ion maps, one might assume that these distribution maps look the same because of the topography of the sample. However, when considering the distribution map of Ag^+^ ions, this theory is proven false. Combining this information with the data obtained from EDX maps, which show a layer of silver and no signal from the support itself except in the spots where the silver layer is broken to reveal the support, give an even better insight into the interaction of silver with the support. It can be stated that the Ag^+^ distribution map shows that the thickness of the layer of silver is not uniform across the grain, but allows Ag^+^ ions to be extracted from the entire surface, whereas Ag^–^ ions are only emitted from the spots of the surface where the silver layer is thin enough to allow the ions from the support to be detected. This could suggest that a Ce–O–Ag bridge or Ce–Ag interaction is responsible for the emission of Ag^–^ ions.

**Figure 10:**
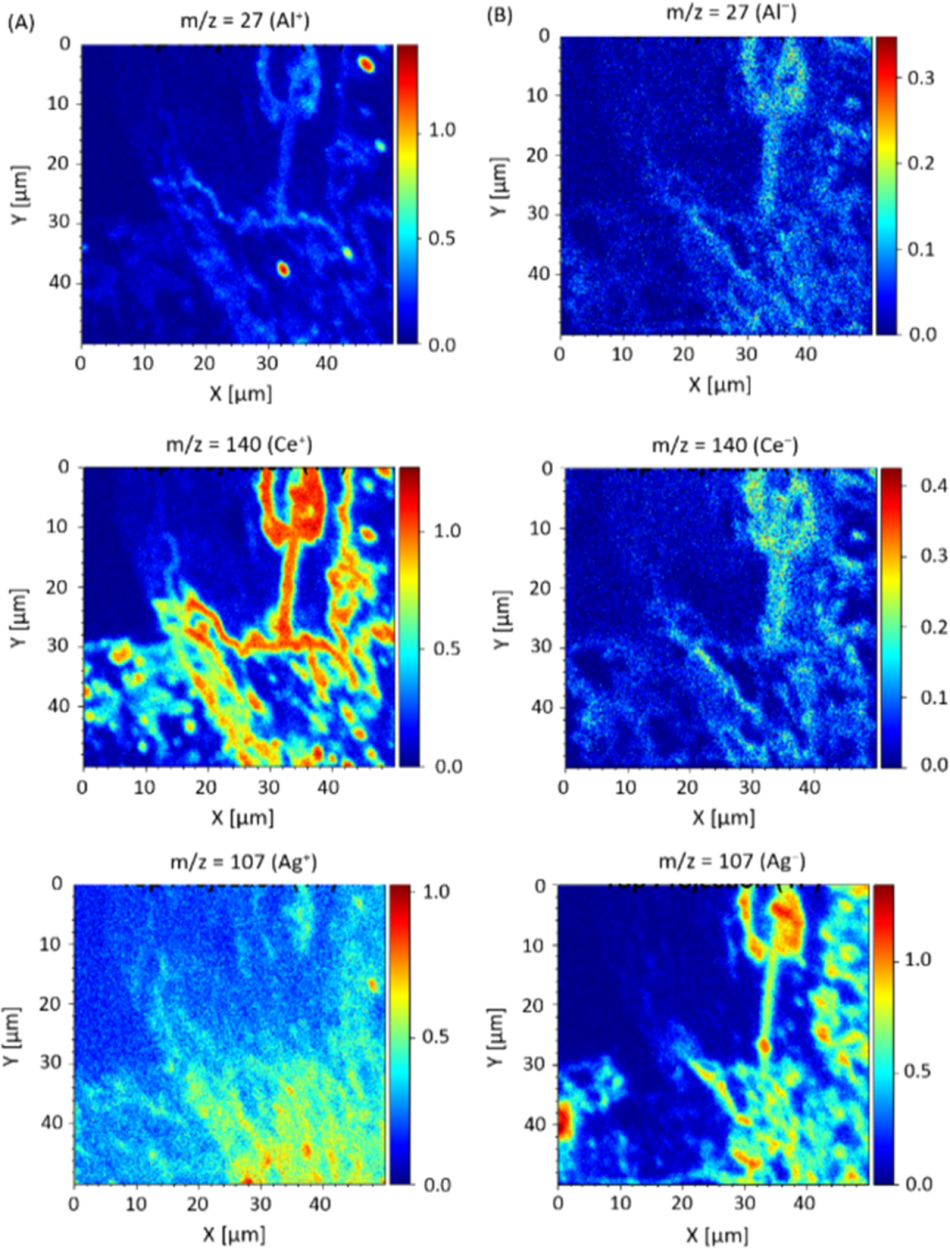
ToF SIMS results: maps of *m*/*z* = 27, 140, and 107 from (A) positive and (B) negative ion spectra.

Three *m*/*z* ratios, namely *m*/*z* = 107 (Ag), *m*/*z* = 40 (Ce), and *m*/*z* = 156 (CeO) from the positive and negative ToF SIMS spectra were normalized to the Ag^+^ or Ag^–^ ion intensity and shown in Figure S12 (Supporting Information). It can be seen that in the positive spectrum, the results are similar for Ag/CeO_2_ and Ag/Ce5Zr (Figure S12A), but significantly different for the other dopants. This indicates that the thicknesses of the silver layer are comparable for these two samples. The Ag^+^/Ce^+^ ratio is approximately 1.0 for these two catalysts, and it is much lower for Ag/Ce5Sr (0.2) and much higher for Ag/Ce5Mg (16.0). The fact that these ratios are very high for Ag/Ce5Mg indicates that the silver layer is the thickest in this catalyst. The high relative abundance of Ce^+^ ions (*m*/*z* = 140) indicates that the silver layer in Ag/Ce5Sr is the thinnest. For Ag/Ce5Al and Ag/Ce10Al, the Ag^+^/Ce^+^ ratios are 2.9 and 2.1, respectively. What is noteworthy and seems more important than the depth of the silver layer is the fact that doping with Al^3+^ impacts the matrix effect and changes the Ce^+^/CeO^+^ ratio with the widest gap of all of the studied supports (Figure S12).

In the negative spectra (Figure S12B), the order of the Ag^–^/Ce^–^ ratio is as follows: Ag/Ce5Al (5.9)> Ag/Ce10Al (7.1) > Ag/CeO_2_ (8.3) > Ag/Ce5Sr (15.1) > Ag/Ce5Zr (15.5) > Ag/Ce5Mg (20.3). The similarity of the values obtained for the two concentrations of Al^3+^ ions, in contrast to catalysts with other dopant ions, shows that it is the type of dopant that is the key parameter. It has to be emphasized that the concentration of cerium ions is the same in all of the catalysts, and so is the silver loading. However, the matrix effect that determines the ease of extraction of ions is substantially changed by the selection of the dopant ion.

## Conclusions

A series of ceria-based solid solutions containing Sr^2+^, Mg^2+^, Al^3+^, or Zr^4+^ was used as silver supports for catalytic soot combustion. Their activity was higher than that of Ag/CeO_2_. The 5% of dopant did not affect the lattice constant of ceria but influenced the properties of the obtained oxide, such as crystallite size, shape of pores, reducibility, etc. As evidenced by EDX maps as well as ToF SIMS maps, the dopant is uniformly spread out in the grains and present in the same spots as the cerium ions. When the loading of the dopant was doubled, the resultant silver catalyst is less active than that supported on the oxide containing 5 atom %. The extent of the effect is dependent on the dopant. It was found that silver in these catalysts was mainly present as a layer (EDX and ToF SIMS maps) of metallic silver (XRD) on the supports. The studies revealed that ToF SIMS is a useful tool in the study of solid oxide solutions and the interaction of the active phase with the supports.

## Supplementary Material



## Notes

Summary of phases and valence state information from different techniques (Table S1); EDX maps of the following supports: Ce5Sr, Ce5Al and Ce5Mg (Figure S1); EDX maps of the following physical mixtures: CeO_2_ + Al_2_O_3_ and CeO_2_ + MgO (Figure S2); EDX maps of the following catalysts: Ag/Ce5Sr and Ag/Ce5Mg (Figure S3); XPS survey spectra of supports: Ce5Al, Ce5Sr, and Ce5Mg and catalysts: Ag/Ce5Al, Ag/Ce5Sr, and Ag/Ce5Mg (Figure S4); Raman measurement results: entire overlay of results obtained for undoped CeO_2_, Ce5Mg, Ce5Zr, Ce5Al, and Ce5Sr, zoom at base of signal peak in the wavenumber range of 700–300 cm^–1^: overlay, and individual samples (Figure S5); XRF spectra of Ag/Ce5Zr and Ag/Ce5Sr (Figure S6); pyridine desorption FTIR results: the entire *v*CCN frequency region after Py desorption at RT, and desorption curves of undoped ceria, Ce5Zr and Ce5Al at different temperatures (Figure S7); results of Hammett indicator tests: colors of supports 24 h after dosing with the indicator solutions (Table S2); thermal analysis results (DTA curves) obtained during tests carried out: in loose contact tests, in tight contact with 5 min-long grinding time and with carbon black as the model soot (tight contact 5 min-long grinding time); *tight contact with 1 min-long grinding, dashed line: uncatalyzed soot combustion *T*_max_ (Figure S8); results of isothermal activity measurements: mass loss curves obtained for selected catalysts with multiple isothermal hold temperatures in one measurement, Ag/Ce5Sr and Ag/Ce5Al with one isothermal hold at 420 °C, and Ag/Ce5Al with a single isothermal hold per measurement at 420 or 440 °C (Figure S9); cyclic test results: DTG curves obtained with reused Ag/Ce5Zr and (B) Ag/Ce10Al (Figure S10); CBS images of Ag/Ce10Al before catalytic tests and after a catalytic test (Figure S11); and ToF SIMS results: relative intensity ratios of Ag, Ce, and CeO ions in positive and negative ion spectra (Figure S12) (PDF)
