# Supplementary material for: When a Low-Temperature Solid Oxide Solution Is the Best Solution: Inserting Guest Ions into the Ceria Host Lattice to Improve Silver Catalyst Activity for Soot Combustion
Source: ACS Appl Mater Interfaces. 2026 Mar 6;18(10):14933–46. doi: 10.1021/acsami.5c21439 (PMC13298811; doi:10.1021/acsami.5c21439)
Supplement: Supplementary file 1 [file am5c21439_si_001.pdf]

Supporting Information

When a low-temperature solid oxide solution is the best solution: Inserting guest ions into the ceria host lattice to improve silver catalyst activity for soot combustion

Ewa M. Iwanek (nee Wilczkowska)

Faculty of Chemistry, Warsaw University of Technology, Noakowskiego 3, 00-664 Warsaw, Poland; ewa.iwanek@pw.edu.pl

Number of pages: 9

Number of figures: 12

Number of tables: 2

Table S1. Summary of Phases and Valence State Information from Different Techniques

Parameter		Ag/CeO_2	Ag/Ce5Sr	Ag/Ce5Zr	Ag/Ce5Mg	Ag/Ce5Al
Number of phases and phase identification (XRD)	Ag	(1): Ag^0 fcc structure	(1): Ag^0 fcc structure	(1): Ag^0 fcc structure	(1): Ag^0 fcc structure	(1): Ag^0 fcc structure
	Ce	(1): CeO_2-type fluorite	(1): CeO_2-type fluorite	(1): CeO_2-type fluorite	(1): CeO_2-type fluorite	(1): CeO_2-type fluorite
	dopant	n.a.	(1): CeO_2-type fluorite	(1): CeO_2-type fluorite	(1): CeO_2-type fluorite	(1): CeO_2-type fluorite
Number of phases (CBS+EDX), other elements detected in phase	Ag	1, none	1, none	1, none	1, none	1, none
	Ce	1, oxygen	1, oxygen, strontium	1, oxygen, zirconium	1, oxygen, magnesium	1, oxygen, aluminum
	dopant	n.a.	1, oxygen, cerium	1, oxygen, cerium	1, oxygen, cerium	1, oxygen, cerium
Oxidation states (XPS)	Catalyst:					
	Ag	Ag^0	Ag^0	Ag^0	Ag^0	Ag^0
	Support:					
	Ce^{3+}/Ce^{4+}	0.18	0.25	0.20	0.23	0.31
	dopant	n.a.	low intensity	Zr^{4+}	low intensity	Al^{3+}

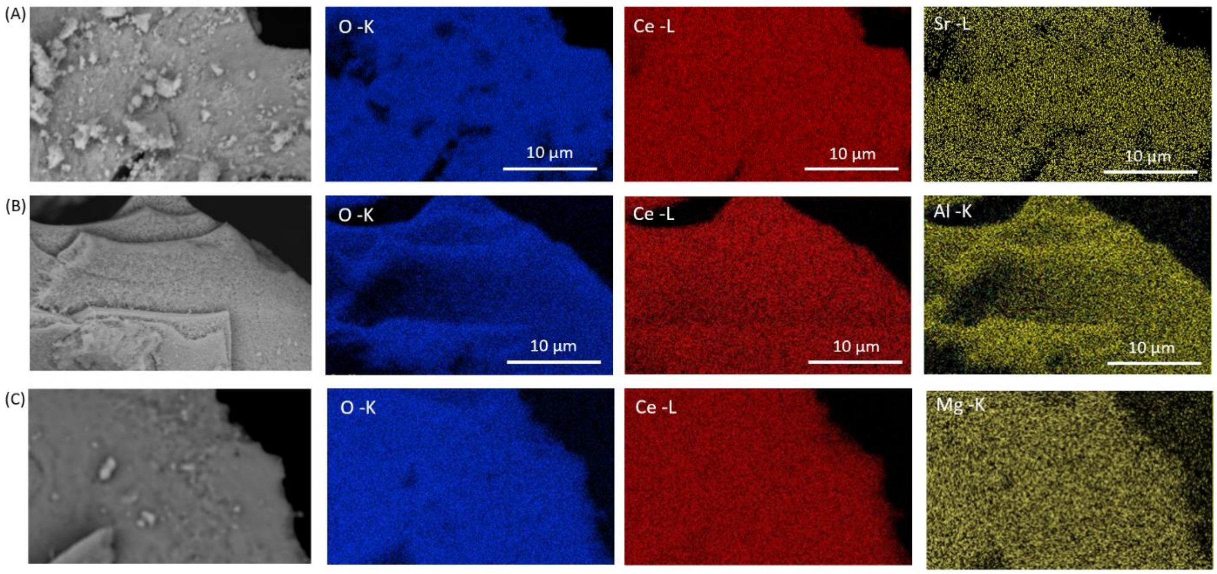

Figure S1. EDX maps of the following supports: (A) Ce5Sr, (B) Ce5Al and (C) Ce5Mg.

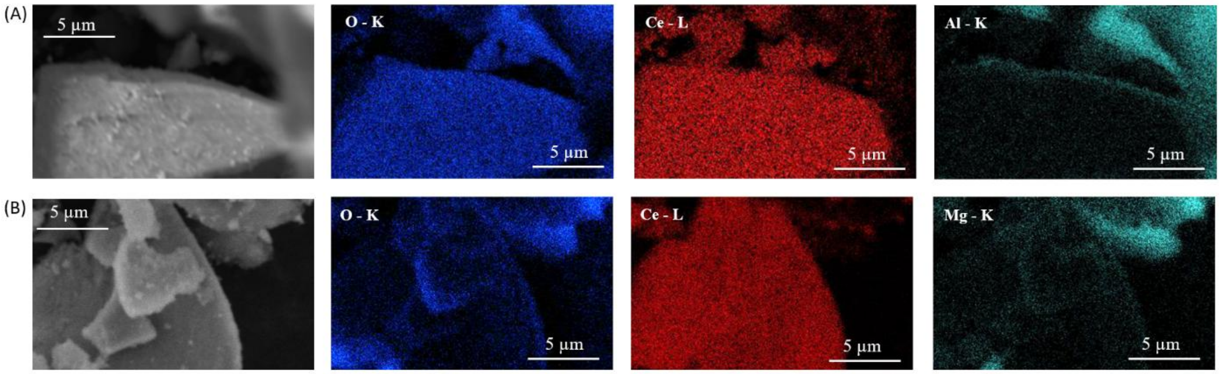

Figure S2. EDX maps of the following physical mixtures: (A) CeO_2+Al_2O_3 and (B) CeO_2+MgO.

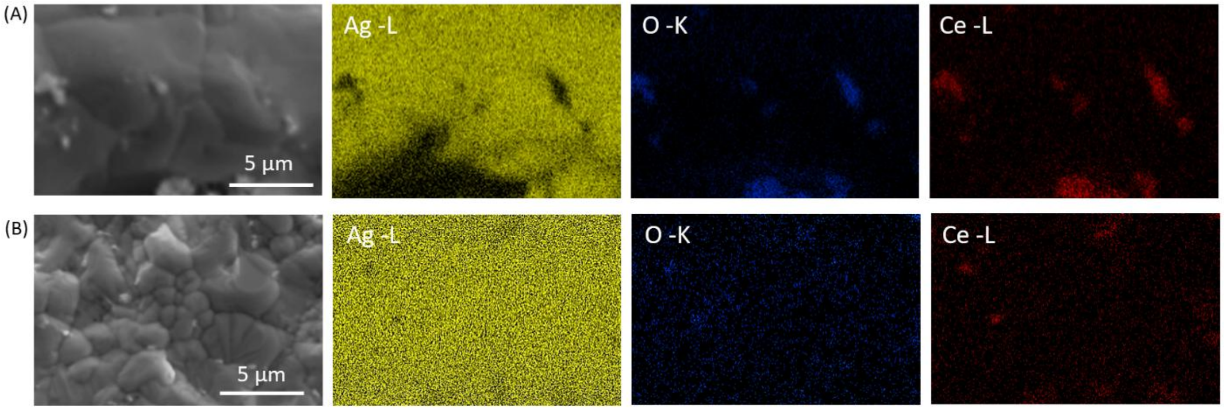

Figure S3. EDX maps of the following catalysts: (A) Ag/Ce5Sr and (B) Ag/Ce5Mg.

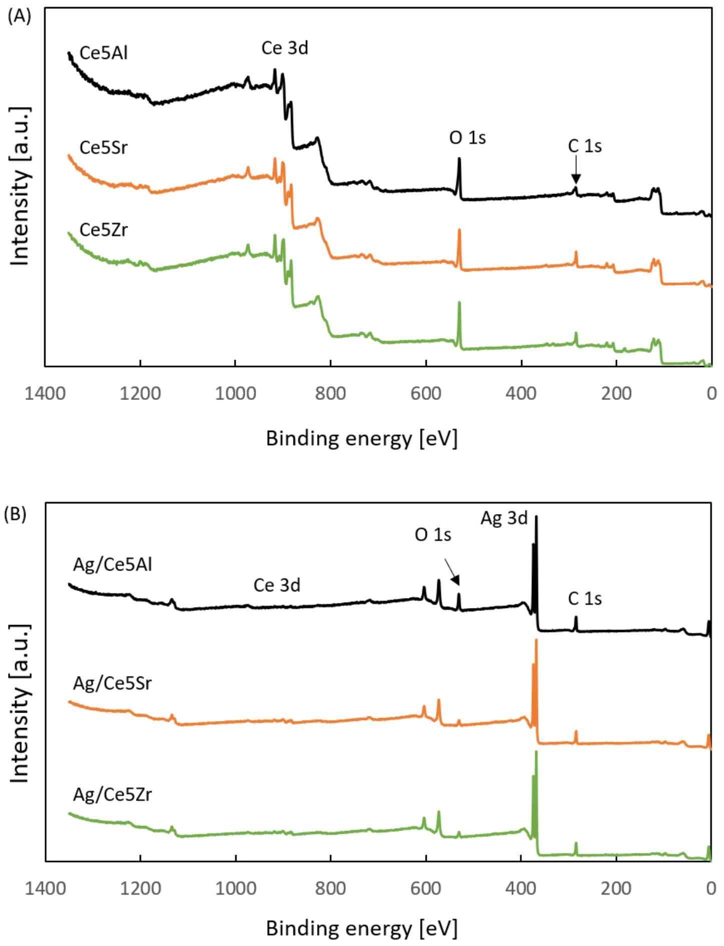

Figure S4. XPS survey spectra of (A) supports: Ce5Al, Ce5Sr and Ce5Mg and (B) catalysts: Ag/Ce5Al, Ag/Ce5Sr and Ag/Ce5Mg.

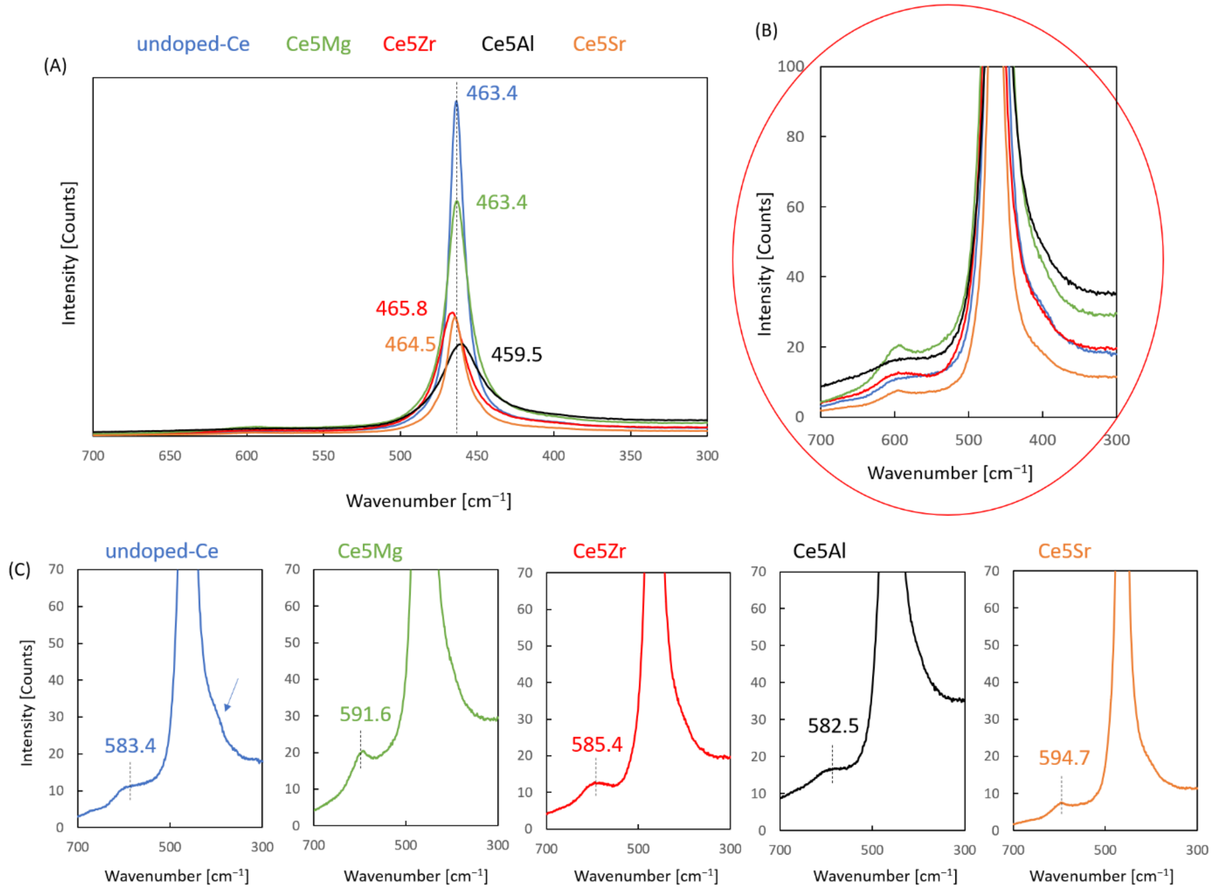

Figure S5. Raman measurement results: (A) entire overlay of results obtained for undoped CeO_2, Ce5Mg, Ce5Zr, Ce5Al and Ce5Sr, (B) zoom at base of signal peak in the wavenumber range of 700-300 cm^{-1}: overlay, and (C) individual samples.

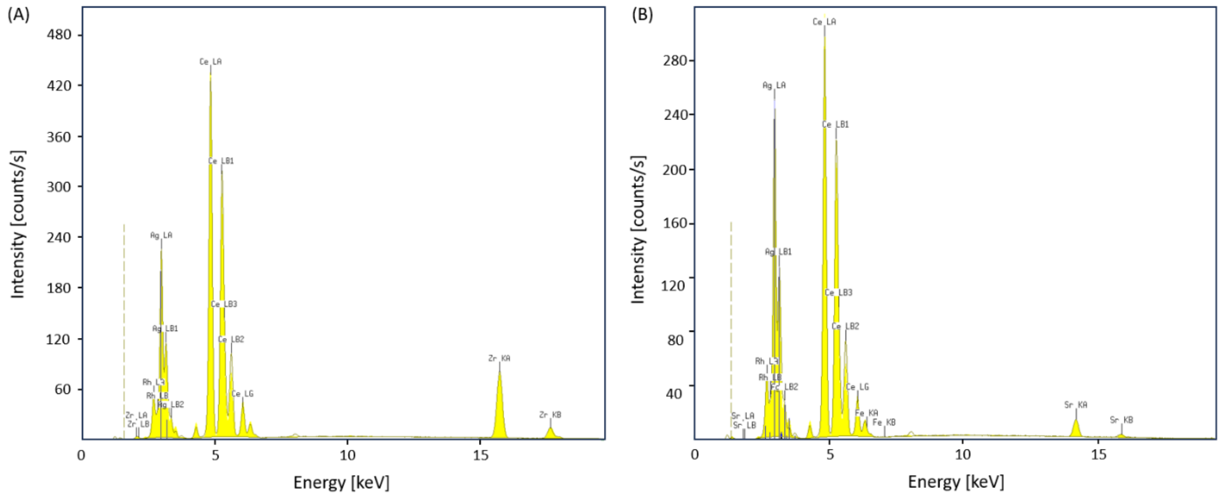

Figure S6. XRF spectra of (A) Ag/Ce5Zr and (B) Ag/Ce5Sr.

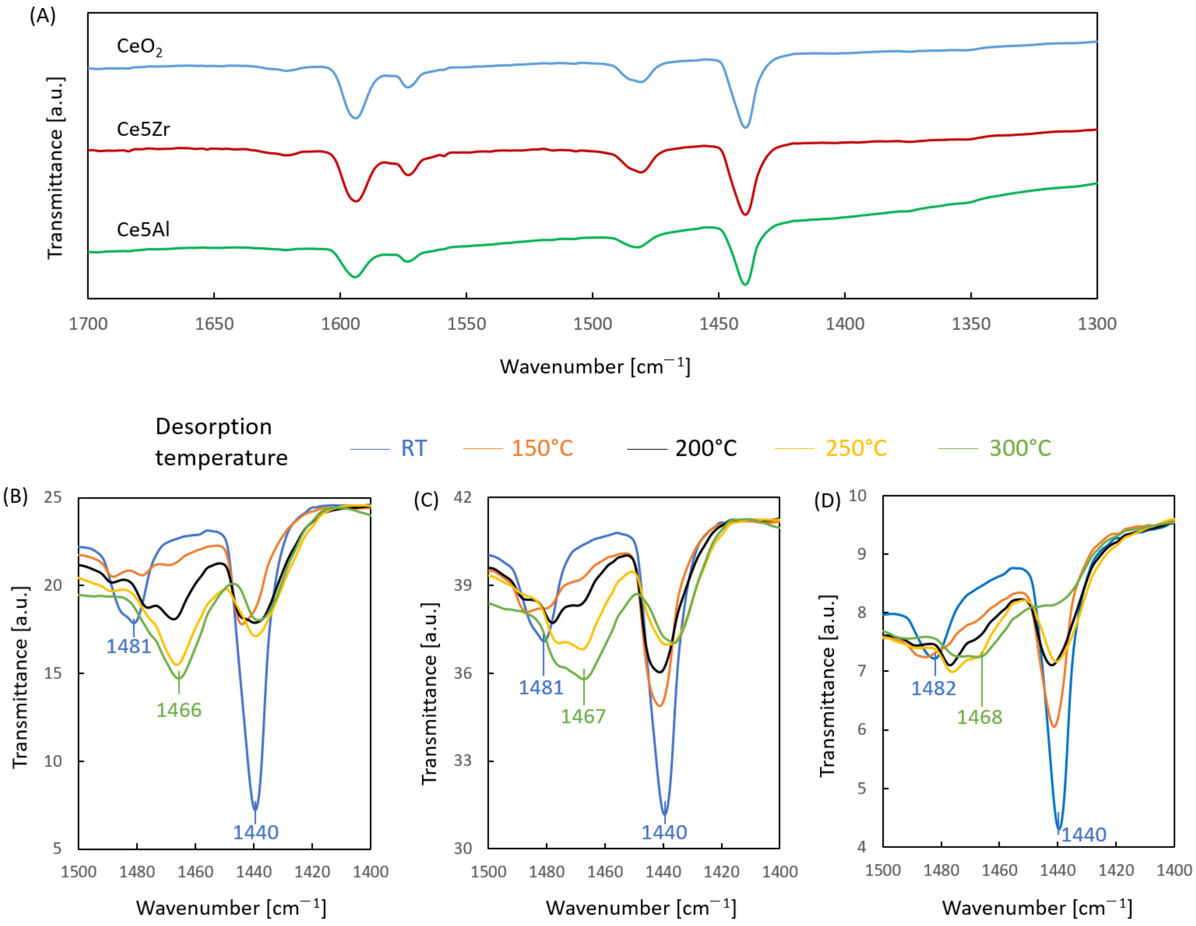

Figure S7. Pyridine desorption FTIR results: (A) the entire νCCN frequency region after Py desorption at RT, and desorption curves of (B) undoped ceria, (C) Ce5Zr and (D) Ce5Al at different temperatures.

Table S2. Results of Hammett Indicator Tests: Colors of Supports 24 hours After Dosing with the Indicator Solutions

pK_{BH+}/pK_A	CeO_2	Ce5Sr	Ce5Mg	Ce5Zr	Ce5Al
22.3	yellow	light brown/reddish	brown/red	reddish	light brown/reddish
18.4	yellow	orange	orange	yellow	yellow
15.0	yellow	orange	red	orange	light orange
9.3	dark pink/violet	purple	violet	purple	violet
7.2	greenish	greenish	greenish	greenish	greenish
4.8	orange	light orange	orange	orange	light orange
0.8	dark blue	light blue	dark blue	light blue	light blue
−3.0	light orange	yellow/orange	yellow/orange	yellow/light orange	yellow
−5.6	yellow	yellow/orange	yellow	yellow	yellow
−8.2	reddish	yellow/pinkish	pinkish/reddish	pinkish	yellow/light orange

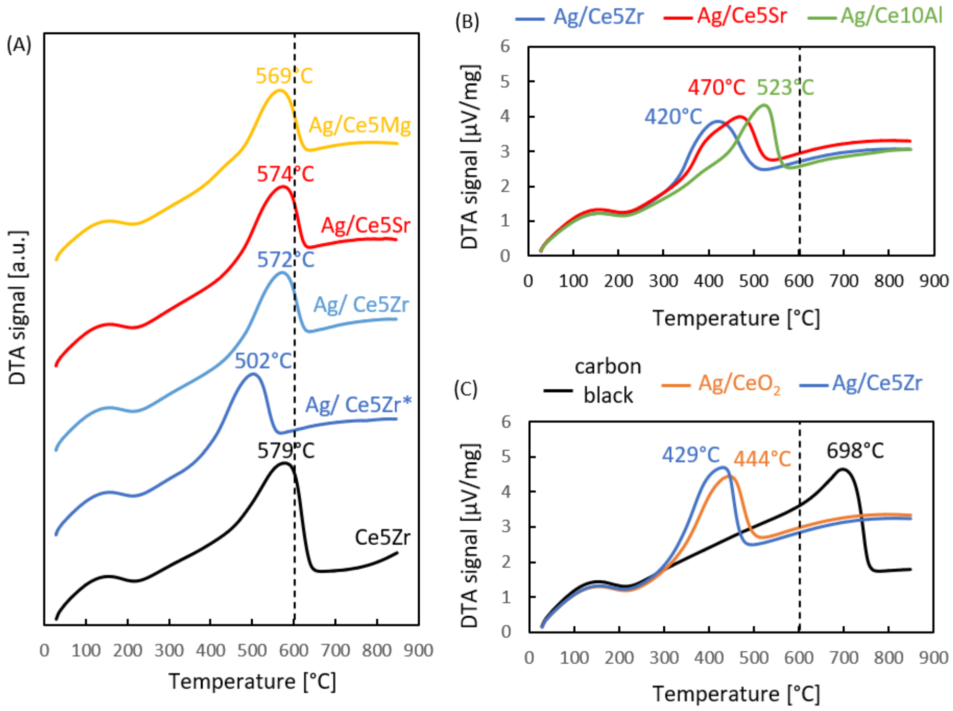

Figure S8. Thermal analysis results (DTA curves) obtained during tests carried out: (A) in loose contact tests, (B) in tight contact with 5-minute-long grinding time and (B) with carbon black as the model soot (tight contact 5-minute-long grinding time); * tight contact with 1-minute-long grinding, dashed line: uncatalyzed soot combustion T_{max}.

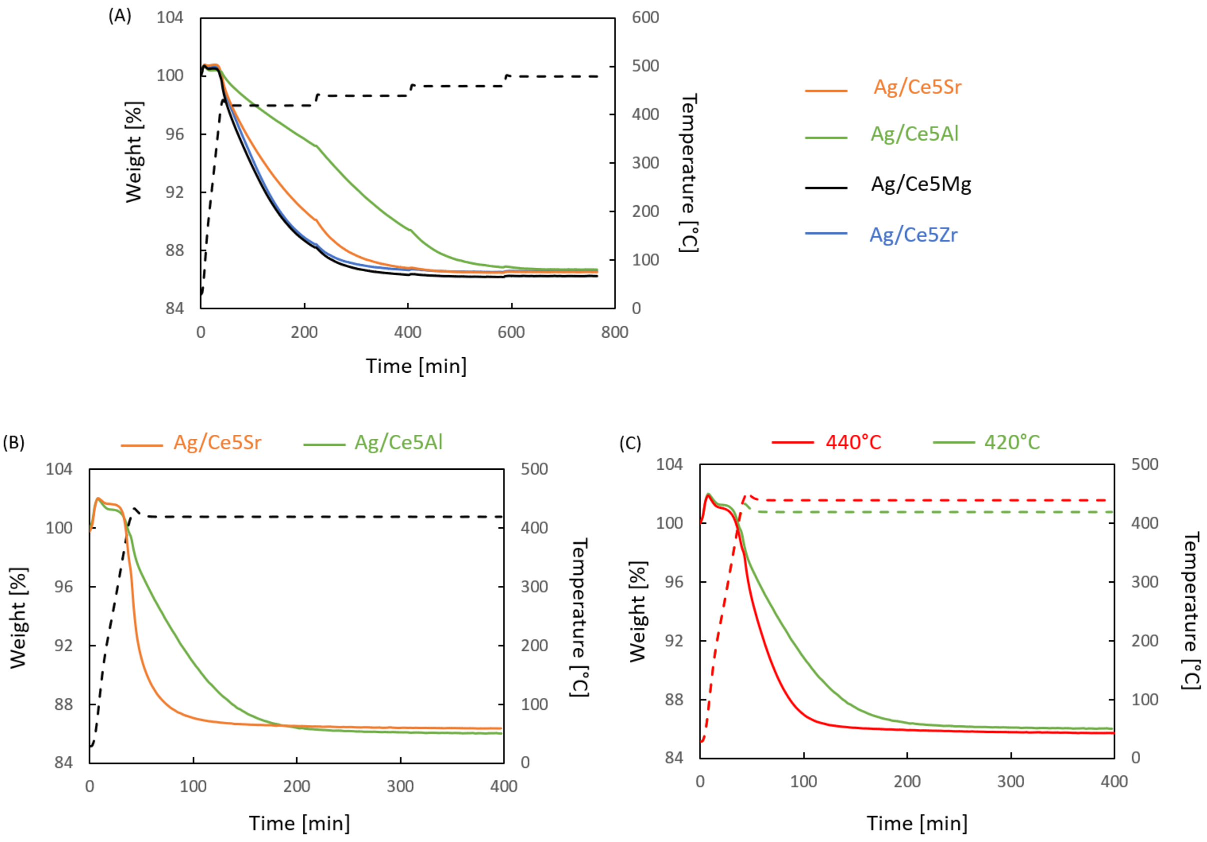

Figure S9. Results of isothermal activity measurements: mass loss curves obtained (A) for selected catalysts with multiple isothermal hold temperatures in one measurement, (B) for Ag/Ce5Sr and Ag/Ce5Al with one isothermal hold at 420°C as well as (C) for Ag/Ce5Al with a single isothermal hold per measurement at 420 or 440°C.

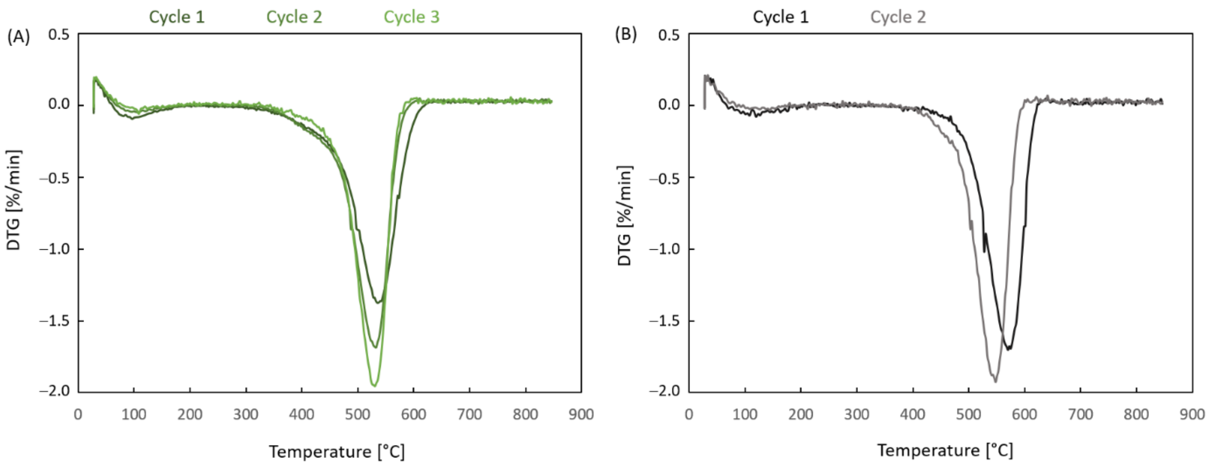

Figure S10. Cyclic test results: DTG curves obtained with reused (A) Ag/Ce5Zr and (B) Ag/Ce10Al.

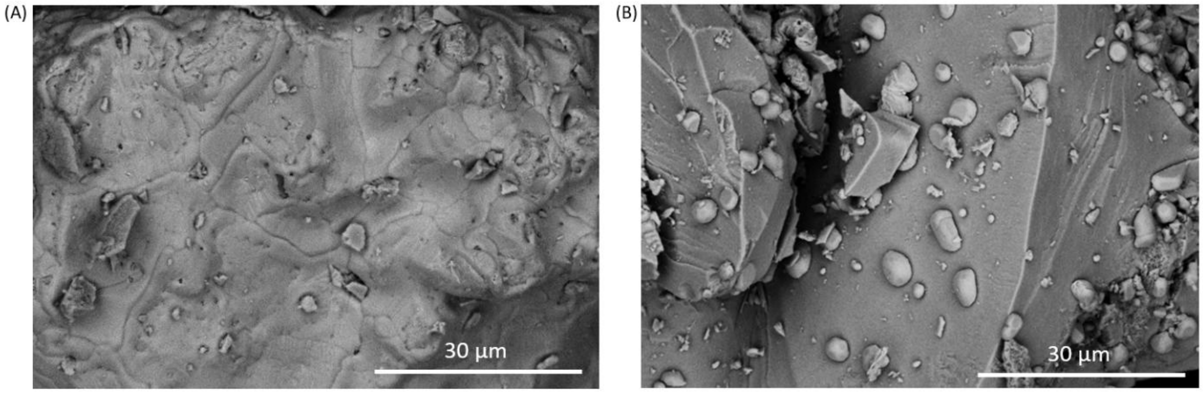

Figure S11. CBS images of Ag/Ce10Al (A) before catalytic tests and (B) after a catalytic test.

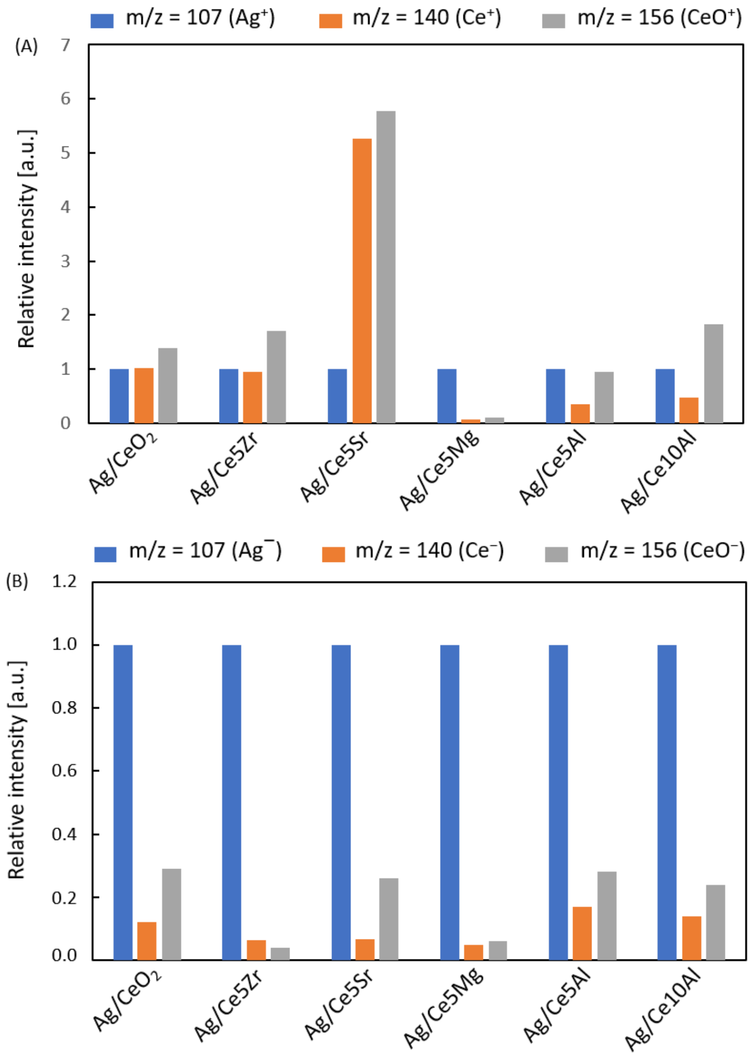

Figure S12. ToF SIMS results: relative intensity ratios of Ag, Ce and CeO ions in (A) positive and (B) negative ion spectra.
